# Transcriptional Profiles of Skeletal Muscle Associated With Increasing Severity of White Striping in Commercial Broilers

**DOI:** 10.3389/fphys.2020.00580

**Published:** 2020-06-16

**Authors:** Yuwares Malila, Tanaporn Uengwetwanit, Sopacha Arayamethakorn, Yanee Srimarut, Krittaporn V. Thanatsang, Francesca Soglia, Gale M. Strasburg, Wanilada Rungrassamee, Wonnop Visessanguan

**Affiliations:** ^1^National Center for Genetic Engineering and Biotechnology (BIOTEC), Thailand Science Park, Pathum Thani, Thailand; ^2^Department of Agricultural and Food Sciences, Alma Mater Studiorum, University of Bologna, Cesena, Italy; ^3^Department of Food Science and Human Nutrition, Michigan State University, East Lansing, MI, United States

**Keywords:** white striping, commercial broiler, gene expression profile, microarray, myopathy

## Abstract

Development of the white striping (WS) abnormality adversely impacts overall quality of broiler breast meat. Its etiology remains unclear. This study aimed at exploring transcriptional profiles of broiler skeletal muscles exhibiting different WS severity to elucidate molecular mechanisms underlying the development and progression of WS. Total RNA was isolated from pectoralis major of male 7-week-old Ross 308 broilers. The samples were classified as mild (*n* = 6), moderate (*n* = 6), or severe (*n* = 4), based on number and thickness of the white striations on the meat surface. The transcriptome was profiled using a chicken gene expression microarray with one-color hybridization technique. Gene expression patterns of each WS severity level were compared against each other; hence, there were three comparisons: moderate vs. mild (C1), severe vs. moderate (C2), and severe vs. mild (C3). Differentially expressed genes (DEGs) were identified using the combined criteria of false discovery rate ≤ 0.05 and absolute fold change ≥1.2. Differential expression of 91, 136, and 294 transcripts were identified in C1, C2, and C3, respectively. There were no DEGs in common among the three comparisons. Based on pathway analysis, the enriched pathways of C1 were related with impaired homeostasis of macronutrients and small biochemical molecules with disrupted Ca^2+^-related pathways. Decreased abundance of the period circadian regulator suggested the shifted circadian phase when moderate WS developed. The enriched pathways uniquely obtained in C2 were RNA degradation, Ras signaling, cellular senescence, axon guidance, and salivary secretion. The DEGs identified in those pathways might play crucial roles in regulating cellular ion balances and cell-cycle arrest. In C3, the pathways responsible for phosphatidylinositol 3-kinase-Akt signaling, p53 activation, apoptosis, and hypoxia-induced processes were modified. Additionally, pathways associated with a variety of diseases with the DEGs involved in regulation of [Ca^2+^], collagen formation, microtubule-based motor, and immune response were identified. Eight pathways were common to all three comparisons (i.e., calcium signaling, Ras-associated protein 1 signaling, ubiquitin-mediated proteolysis, vascular smooth muscle contraction, oxytocin signaling, and pathway in cancer). The current findings support the role of intracellular ion imbalance, particularly Ca^2+^, oxidative stress, and impaired programmed cell death on WS progression.

## Introduction

White striping (WS) abnormality, as depicted by the appearance of white lines running parallel to muscle fibers on the surface of chicken breast meat, has become of great concern in the poultry industries. Based on their aberrant visual characteristics, the breasts with severe WS condition have reduced appeal to consumers and are downgraded for use as processed meat products, thus resulting in significant economic losses for the poultry industry (Kuttappan et al., 2012, 2016; Petracci et al., 2014). The WS breasts consistently exhibit technological challenges, particularly inferior water holding capacity (Kuttappan et al., 2013; Petracci et al., 2013). The nutritional profile of the WS meat is shifted toward higher fat and lower protein proportions compared to non-WS chicken breasts (Petracci et al., 2014; Zambonelli et al., 2016; Malila et al., 2018).

The specific etiology of the WS abnormality is still under investigation. In order to elucidate the molecular mechanisms underlying development of WS myopathy, Zambonelli et al. (2016) compared the gene expression patterns of pectoralis major muscle from Ross 708 broiler hybrids exhibiting severe WS together with wooden breast (WB) against those without any macroscopic lesions. The differentially expressed genes (DEGs) identified were associated with muscle development, inflammation, polysaccharide metabolism, and calcium signaling. It is worth noting that, in the study of Zambonelli et al. (2016), the abnormal group was affected with both WS and WB conditions. Therefore, the biological pathways directly related with WS development alone could be diluted by the strong effect of WB myopathy and might not be clearly shown in the results. Transcriptome profiles of severe WS (white line thickness >1 mm) in pectoralis major collected from 42-day-old Cobb 500 broilers showed significant increases in transcripts involved in the activation of hypoxia, immune responses, and angiogenesis compared with that of nondefective samples (Marchesi et al., 2019). Dysregulated metabolic processes were also identified at the metabolomic level (Boerboom et al., 2018) with opposite directionalities of some intermediates of tricarboxylic acid cycle as well as incomplete breakdown products of protein catabolism. Recently, using droplet digital polymerase chain reaction (ddPCR), an increased absolute transcript abundance of hypoxia-inducible factor 1 alpha (*HIF1A*) along with differential expression of *HIF1A*-related genes were identified in pectoralis major of the WS- and WS+WB-affected Ross 308 broilers (Malila et al., 2019). So far, the growing evidence is pointing toward oxidative stress as a primary contributor to development of WS muscle.

While the previous studies focused on exploring different transcriptomic or metabolomic profiles of severe WS in respect to nondefective muscles, the molecular aspects underlying the progression in severity of WS lesion still remain unclear. The objective of this study was to examine differences in transcriptome profiles and define molecular mechanisms associated with elevating WS severity in pectoralis major collected from 7-week-old male commercial Ross 308 broilers. Our findings regarding the progression of the WS myopathy at molecular level provides complementary information to existing studies concerning the development of WS in commercial broilers. The current findings could lay foundation in the development of an intervention to prevent or delay the progression of WS abnormality.

## Materials and Methods

### Samples and Sample Collection

All breast samples were collected within 20 min postmortem from 83 broiler carcasses (7-week-old male Ross 308), raised at a facility of a local poultry meat producer and processed in the industrial processing plant under the routine Halal standard practice. One side of each breast was immediately dissected, snap-frozen, and kept in liquid nitrogen during transportation back to the laboratory (Pathum Thani, Thailand). The muscle samples were stored at -80°C until total RNA isolation. The other side of the breast was dissected, placed in a plastic bag, kept on ice during transportation, and stored at 4°C until use.

In this study, all samples were purchased in the form of whole carcasses from the commercial processing plant. Neither experimental treatments or scientific procedures were subjected to the living animals. Thereby, according to BIOTEC Institutional Animal Care and Use Committee, the ethical approval was not required.

### White Striping Defect Classification

**FIGURE 1:**
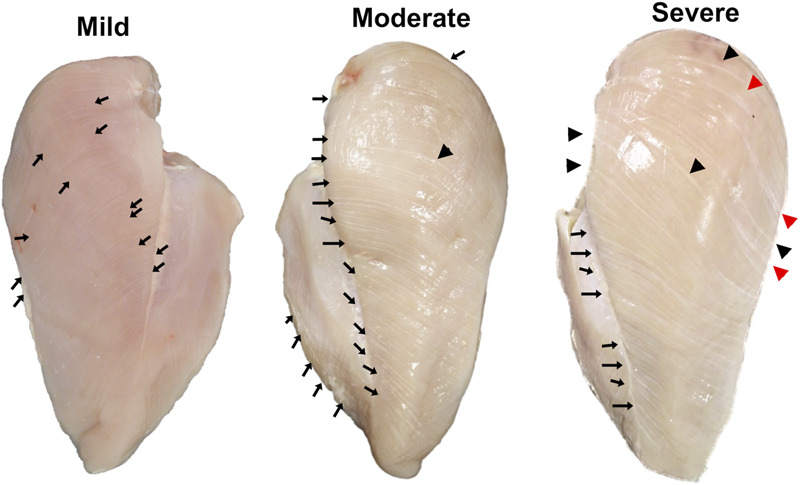
Classification of white striping (WS) abnormality by degree of severity. Mild, 1–40 white lines with the thickness of ≤0.5 mm; moderate, more than 40 white lines or 1–5 line(s) with the thickness of 1–1.9 mm; and severe, more than 5 lines with the thickness of 1–1.9 mm thickness or at least 1 white line with thickness >2 mm. Black arrows, black triangles, and red triangles indicate white lines with thickness <1.0 mm, 1.0–1.9 mm, and ≥2.0 mm, respectively.

The WS abnormality and its severity were inspected using the criteria previously described (Malila et al., 2018). Three WS severity levels (Figure 1), including “mild WS” (1–40 white lines with the thickness of ≤0.5 mm), “moderate WS” [more than 40 white lines or 1–5 line(s) with the thickness of 1–1.9 mm], and “severe WS” (more than 5 lines with the thickness of 1–1.9 mm thickness or at least 1 white line with thickness >2 mm), were observed among the samples. The samples showing other abnormalities, e.g., WB, were excluded from this experiment in order to avoid any confounding interpretations from such myopathies. Accordingly, 29 mild, 38 moderate, and 4 severe WS samples were available for this study. Six biological replicates were randomly selected from “mild” and “moderate” groups. For “severe” samples, all four samples were used. Although our study was initially designed to include the analysis of non-WS samples, there were no nondefective samples (without any macroscopic trait associated with the development of WS) found among our total of 83 samples of the 7-week-old broilers collected for this trail. This phenomenon emphasized the high prevalence of WS abnormality in 7-week-old commercial broilers.

### Total RNA Isolation

Total RNA was isolated using TRIzol^TM^ Reagent (Invitrogen), subsequently treated with DNase I (Thermo Scientific, Inc.), and repurified using GeneJET RNA Cleanup and Concentration Micro Kit (Thermo Scientific, Inc.). The samples with an RNA integrity number exceeding 7.0 were analyzed by microarray hybridization at Molecular Genomics Pte. Ltd. (Singapore) and quantitative real-time polymerase chain reaction (qPCR).

### Microarray Platform and Experimental Design

The Agilent SurePrint G3 Custom GE 8 × 60K chicken gene expression microarray (Agilent Technologies, Inc.) used in this study was designed based on the National Center for Biotechnology Information (NCBI) *Gallus gallus* Annotation Release 103. The detailed information of the array is available at NCBI Gene Expression Omnibus (GEO) with the platform accession GPL24307.

**FIGURE 2:**
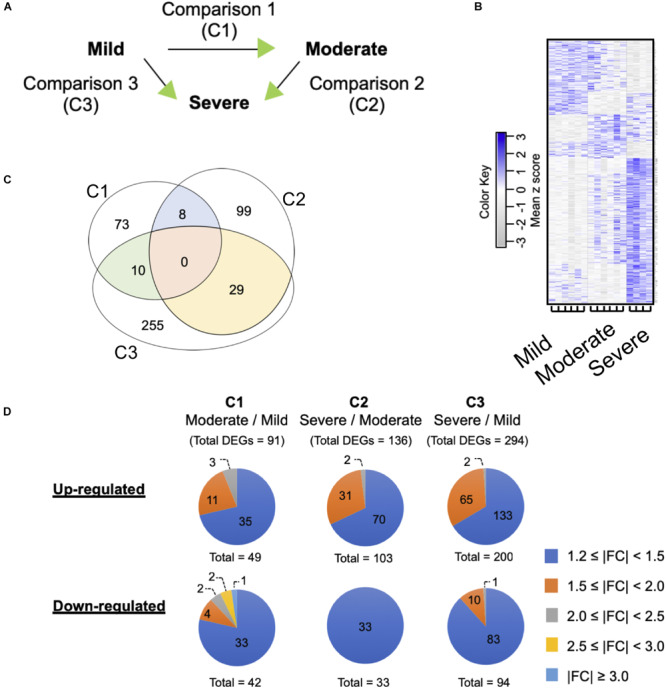
Differential gene expression associated with white striping (WS) severity. **(A)** Microarray experimental design. The green arrow heads indicate that the particular WS severity level was the numerator in such comparison. **(B)** Heatmap gene cluster classification for mild (*n* = 6), moderate (*n* = 6), and severe (*n* = 4) WS samples. The expression of each gene is presented in the rows. **(C)** Venn diagram depicts number of differentially expressed genes (DEGs) obtained from each comparison. The criteria used in identification of DEGs is a combination between false discovery rate ≤0.2 and absolute fold change (|FC|) ≥ 1.2. **(D)** Numbers of up- and downregulated DEGs clustered based on |FC|.

To determine differential gene expression associated with WS severity, expression patterns obtained among the WS levels were compared against one another (Figure 2A). Overall, 16 arrays were hybridized in order to accomplish three comparisons (C1, moderate against mild; C2, severe against moderate; C3, severe against mild).

### Microarray Hybridizations

Total RNA was labeled using one-color low-input Quick Amp labeling kit (Agilent) following the company’s instruction. In brief, 100 ng total RNA was reverse transcribed into double-stranded complementary DNA (cDNA) by priming with oligo(dT) primer. The synthesized cDNA was subjected to an *in vitro* transcription using T7 RNA polymerases to produce cyanine 3-CTP-labeled complementary RNA (cRNA). After purification, 600 ng of the purified cRNA was hybridized onto the Agilent SurePrint array at 65°C for 17 h. The array was washed, gently blotted dry, and scanned using an Agilent High-Resolution Microarray Scanner (C Model, Agilent). The TIFF image was saved and analyzed using Agilent Feature Extraction Software version V10.7.1.1 (Agilent).

### Microarray Data Analysis and Gene Annotation

Raw microarray signal values were generated by Agilent Feature Extraction Software version V10.7.1.1 (Agilent). Feature extraction and probe quality control were processed using GeneSpring software. The data were further analyzed in R version 3.4.0. The signal values of probes deemed to be suspicious or faulty were quarantined using flags and expression values. Flags were categorical indicators including “detected,” “compromised,” and “not detected” from the scanner. Probes that were flagged as “not detected” and “compromised” were assigned to not available values (NA). Raw signal intensities of probes below 20 for that microarray were assigned as NA values. Probes with missing intensity data were discarded. The filtered raw data were normalized in R using quantile normalization (Bolstad, 2017) and using combat normalization afterward (Müller et al., 2016; Leek et al., 2017). Log_2_ transformation was applied to normalized data for statistical analysis. Statistically significant difference of each transcript between two treatments was identified by analysis of independent two-group *t* test. The DEGs were identified using combined criteria of false discovery rate (FDR) ≤ 0.05 and absolute fold change (|FC|) ≥ 1.2. Positive and negative FC values represent increased or decreased expression of a particular gene in the samples exhibiting a greater severity level relative to its counterparts. All microarray data have been submitted to the NCBI GEO repository with GEO accession number GSE107362.

### Confirmation of Differential Gene Expression

Twenty-one genes, resulting in a total of 63 comparison counterparts, were chosen for confirming the microarray data. Of the 63 comparison counterparts, 11 and 18 showed decreased and increased expressions, respectively, with |FC| ≥ 1.2, whereas the rest of the genes exhibited |FC| < 1.2. Primers (Supplementary Table S1) were designed using Primer-BLAST^1^.

Changes in expression patterns of the selected genes observed in the current microarray analysis were confirmed using qPCR (Malila et al., 2015). Threshold cycle (Ct) was analyzed using BioRad CFX Manager 2.1 software (BioRad). Hypoxanthine-guanine phosphoribosyltransferase (*HPRT*) was chosen as the reference gene due to its unchanged expression across WS groups. Relative messenger RNA (mRNA) abundance of each gene in the sample group relative to its designated counterparts was calculated based on 2^–ΔΔ*Ct*^ method (Livak and Schmittgen, 2001). To determine statistical difference between two treatments, ΔCt was used in calculation using Student’s *t* test. The significance was set at *p* value of 0.05. All relative abundances obtained from qPCR were plotted against log_2_FC analyzed from microarrays.

### Biological Function and Pathway Analysis of Differentially Expressed Genes

All DEGs of each comparison were grouped into known networks and pathways using Ingenuity Pathway Analysis^®^ (IPA) (trial version, Qiagen) and Kyoto Encyclopedia of Genes and Genomes (KEGG) pathway (Kanehisa et al., 2012). Mapping takes the identifiers from the DEGs and compares them to the database. In IPA, the selected DEGs were mapped to the Ingenuity Knowledge Base database and were then ranked by score, which is calculated based on the hypergeometric distribution and Fisher’s exact test. The significance was set at a *p* value of 0.05. The enrichment of genes based on KEGG pathways was the number that has highest number of genes mapped on the pathway.

## Results

### Differentially Expressed Genes Associated With Increasing WS Severity

The current microarray analysis revealed 91, 136, and 294 differentially expressed genes in C1, C2, and C3 comparisons, respectively (Figures 2B,C). The complete DEGs lists for each comparison are available in Supplementary Table S2. No unique genes were common to all three comparisons. However, C1 shared 8 and 10 DEGs with C2 and C3, respectively; for C2 and C3, there were 29 common DEGs (Supplementary Table S3). Approximately 75% of the DEGs in all comparisons showed |FC| ranging between 1.2 and 1.5 (Figure 2D), except for the C2 of which all 33 downregulated DEGs fell within such |FC| range. Numbers of the up- and downregulated transcripts were comparable within each comparison. The most up- and downregulated genes in C1 were chromosome 3 open reading frame (*C1ORF95*, FC = 2.2) and nucleolin-like (LOC107057290, FC = -3.3), respectively. Mitochondrial translational factor 2 (*MTIF2*, FC = 2.3) and poly(A)-specific ribonuclease regulatory subunit (*PAN3*, FC = -9.6) were the most changed DEGs in C2. For C3, general transcription factor IIF subunit 1-like (LOC107051030, FC = 2.1) and neuronal pentraxin 2 (*NPTX2*, FC = -2.5) were the DEGs showing the largest changes.

**FIGURE 3:**
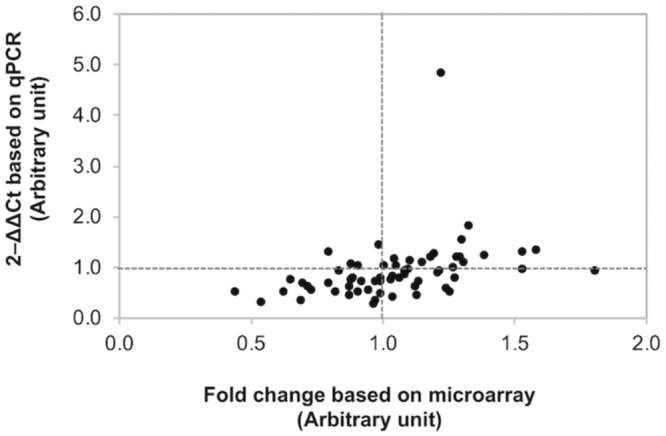
Confirmation of differential gene expression. Scatter plot illustrates the fold changes of 21 selected genes determined by microarray and quantitative real-time PCR (qPCR). Of the 63 comparisons, 45 counterparts (accounted for 71.4%) show comparable results between microarray and qPCR.

The changes in gene expression patterns from the present microarray data were further validated using qPCR (Figure 3). Of the 63 comparison counterparts, the changes in expression of 45 counterparts accounted for 71% of the qPCR data, exhibiting similar trends as that acquired from the microarray platform. As expected, the |FC| values obtained from qPCR of some genes were greater than those reported in microarray analysis. This is due mainly to better sensitivity of qPCR over microarray (Camarillo et al., 2011).

### Functional and Pathway Analysis

In order to review the enriched biological functions, the DEGs were analyzed using a trial version of IPA. Based on the IPA knowledge base, 50 (55%), 89 (65%), and 191 (65%) DEGs of C1, C2, and C3, respectively, were recognized and mapped into the respective canonical pathways. Considering the top altered physiological functions (Table 1), the increment of WS severity from mild to moderate (C1) and from moderate to severe (C2) similarly impacted organ morphology and development as well as nervous system function. On the other hand, in C3, the large abundance of DEGs involved cardiovascular system and connective tissue. For cellular functions (Table 2), cell-to-cell signaling and interaction was the overlapping category among the three comparisons. The increasing WS severity from mild to moderate (C1) mainly affected amino acid, carbohydrate, and lipid metabolisms. Although the altered cellular functions in C2 were somewhat similar with those in C1, the DEGs involved in cellular morphology and cellular assembly and organization were also enriched. As for C3, the large enrichment of DEGs was related with cellular compromise and the changes in cellular growth, proliferation, and cell cycle. All DEGs in the top altered cellular functions are listed in Supplementary Table S3.

**TABLE 1:** The altered physiological functions associated with increasing white striping severity.

**Physiological functions**	***P* value range**		**#molecules**
**C1 (Moderate/Mild)**			
Organ morphology	4.59E-02	9.12E-04	6
Visual system development and function	9.12E-04	9.12E-04	2
Nervous system development and function	4.39E-02	2.13E-03	6
Organismal development	3.15E-02	2.13E-03	7
Skeletal and muscular system development and function	5.00E-02	2.13E-03	4
**C2 (Severe/Moderate)**			
Nervous system development and function	3.84E-02	2.97E-04	13
Tissue morphology	3.49E-02	2.97E-04	17
Cardiovascular system development and function	3.74E-02	2.30E-03	6
Organ morphology	3.84E-02	2.30E-03	14
Organismal development	3.84E-02	2.30E-03	19
**C3 (Severe/Mild)**			
Cardiovascular system development and function	1.68E-02	7.09E-05	18
Organismal development	1.68E-02	7.09E-05	30
Tissue morphology	1.68E-02	7.09E-05	32
Nervous system development and function	1.68E-02	2.90E-04	29
Connective tissue development and function	1.68E-02	4.21E-04	10

**TABLE 2:** The altered cellular functions associated with increasing white striping severity.

**Cellular functions**	***P* value range**		**#molecule**
**C1 (Moderate/Mild)**			
Amino acid metabolism	3.77E-02	1.20E-03	3
Small molecule biochemistry	5.00E-02	1.20E-03	13
Carbohydrate metabolism	5.00E-02	1.40E-03	5
Lipid metabolism	4.59E-02	1.40E-03	3
Cell-to-cell signaling and interaction	5.00E-02	2.13E-03	6
**C2 (Severe/Moderate)**			
Cell morphology	3.91E-02	2.97E-04	18
Protein synthesis	3.84E-02	8.10E-04	6
Cell-to-cell signaling and interaction	3.46E-02	3.31E-03	11
Cellular assembly and organization	3.91E-02	3.31E-03	18
Carbohydrate metabolism	3.08E-02	3.90E-03	9
**C3 (Severe/Mild)**			
Cellular compromise	1.68E-02	4.21E-04	14
Cellular morphology	1.68E-02	5.19E-04	30
Cellular growth and proliferation	1.22E-02	6.20E-04	20
Cell-to-cell signaling and interaction	1.68E-02	8.58E-04	25
Cell cycle	1.68E-02	1.04E-03	13

**FIGURE 4:**
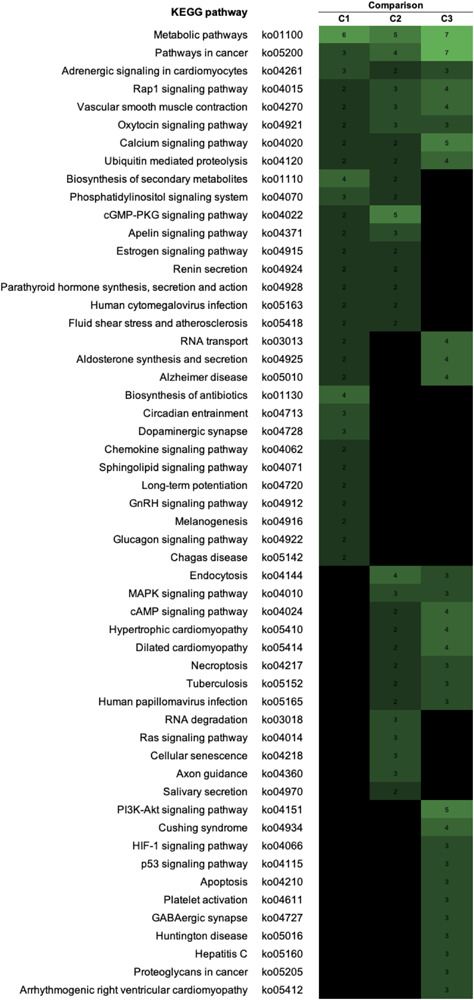
The enriched biological pathways associated with white striping (WS) severity. Heatmap depicts the 30 enriched altered Kyoto Encyclopedia of Genes and Genomes (KEGG) pathways in each comparison. C1, moderate WS/mild WS; C2, severe WS/moderate WS; C3, severe WS/mild WS. The darker color indicated fewer numbers of differentially expressed genes mapped into the pathway.

**FIGURE 5:**
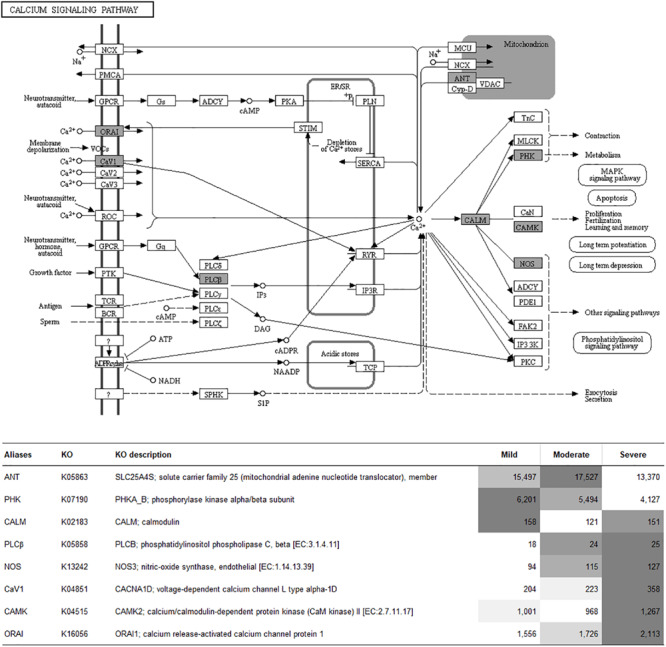
Calcium signaling pathway. The proteins highlighted in gray indicate the differential expression of their encoded genes in at least one comparison. The table indicates fluorescent signal intensity obtained from microarray analysis.

**FIGURE 6:**
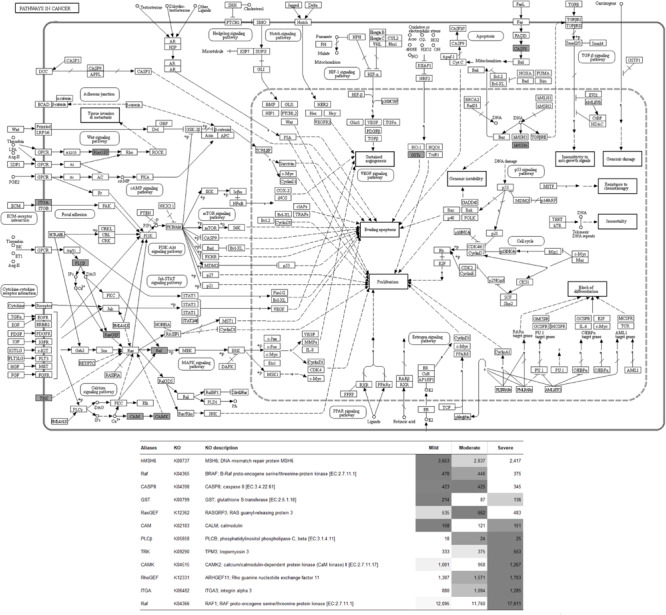
Pathway in cancer. The proteins highlighted in gray indicate the differential expression of their encoded genes in at least one comparison. The table indicates fluorescent signal intensity obtained from microarray analysis.

To visualize the functional interactions among the DEGs, all DEGs of each comparison were subsequently submitted to KEGG pathway database. The top 30 enriched pathways annotated by KEGG are depicted in Figure 4. Among those enriched pathways, eight pathways, including metabolic pathways, calcium signaling (Figure 5), pathway in cancer (Figure 6), ubiquitin-mediated proteolysis (Supplementary Figure S1), RAP1 signaling (Supplementary Figure S2), adrenergic signaling in cardiomyocytes (Supplementary Figure S3), vascular smooth muscle contraction (Supplementary Figure S4), and oxytocin signaling (Supplementary Figure S5) are overlapped among all severity levels. The altered pathways uniquely defined for each comparison and the overlapping pathways are discussed in detail below.

## Discussion

The severity of the WS abnormality varies among the meat depending on the age, the gender, and the size of the chickens as well as depending on their management, e.g., feeding regime. In our previous investigation (Malila et al., 2018), the incidence of the WS abnormality was as high as 98% of the samples with broad spectrum of severity. The majority of the abnormal meat samples were categorized as mild (55.7% of 183 collected broilers) which showed little to no effect on both nutritional and technological properties of the meat. In contrast, severe WS was detected at a small prevalence of 3.3% but significantly impact the quality of the resulting meat (Malila et al., 2018). Once downgraded and used for manufacturing processed meat products, the severe WS breasts, even in small proportions, might alter the quality and consistency of an entire batch of processed products (Petracci and Cavini, 2012). In this study, we profiled transcriptomes within the pectoralis major of 7-week-old broilers exhibiting three different WS levels (i.e., mild, moderate, and severe) with the aim of understanding how changes in the transcriptome are associated with increasing WS severity. Since we did not obtain any nondefective breast samples from our sampling sites, the experiment was modified to compare each pair of defective based on their WS severity (Figure 2A).

Regarding the shared DEGs between each comparison pair (Supplementary Table S3), the eight shared DEG responses between C1 and C2 exhibited comparable fold changes but in opposite directions. The results indicated that differential gene expression patterns were associated with the progression of the myopathic conditions from mild to moderate, followed by a return to non-significant levels similar to those of the mild samples when the WS severity increased from moderate to a severe degree. These expression patterns suggest the crucial roles of those DEGs in development of moderate WS as well as the potent metabolic switch when the myopathy progressed from moderate to severe level. Two out of the eight shared DEGs, the transcripts of dimethyladenosine transferase 1 (*DIMT1*) and zinc finger protein 251 (*LOC101750329*), are involved in RNA processing. The other two transcripts, the platelet-specific isoform of phosphofructokinase (*PFKP*) and phytanoyl-CoA dioxygenase domain-containing protein 1 (*LOC100858951*), play essential roles in carbohydrate and lipid metabolisms, respectively. The gene *CALM* encodes the Ca^2+^-binding protein calmodulin that mediates a wide range of cellular response to the changes in intracellular [Ca^2+^] (Hamilton et al., 2000). The relevant roles in maintenance of cellular structure of mitochondrial ribosome-associated GTPase 2 (*mtg2*) and spastin (*SPAST*) have been documented (Datta et al., 2005; Roll-Mecak and Vale, 2008). For eva-1 homolog B (*EVA1B*), although the specific function of this member of EVA1 family is still scarce, the roles of EVA1 in mediating muscle cell extensions were previously demonstrated in the nematode *Caenorhabditis elegans* (Chan et al., 2014).

Based on the fold changes of the 10 DEGs shared between C1 and C3, the expressions of those DEGs were altered when the WS severity progressed from mild to the other severity degrees. Among those 10 shared DEGs, increased expressions of prolyl 4-hydroxylase, transmembrane (*P4HTM*), and sperm autoantigenic protein 17 (*SPA17*) were of interest. The *P4HTM* encodes an endoplasmic reticulum-anchored prolyl 4-hydroxylase possessing a transmembrane domain. Unlike the other prolyl 4-hydroxylases that act on collagens, this specific isoform catalyzes hydroxylation of the critical prolines in HIF-1α but not in type I procollagen, suggesting the crucial participation of this isoform in adaptive response under reduced oxygen availability (Koivunen et al., 2007). Transcription of this gene was induced in human cell lines exposed to hypoxic condition (Koivunen et al., 2007), supporting the greater extent of hypoxia within the moderate and severe WS breasts in comparison to mild WS. As for *SPA17*, the potential pathogenic role of this transcript, but not the protein, in highly proliferating and neoplastic cells has been addressed (De Jong et al., 2002). The increased *SPA17* mRNA abundance in moderate and severe WS might reflect increased cell proliferation within those samples. Considering the DEGs shared between C2 and C3, similar fold changes between those two comparisons indicated the upregulation or, in the case of neuronal pentraxin 2 (*NPTX2*), downregulation only in severe WS samples. Among those 29 shared DEGs between C2 and C3, six transcripts were annotated as non-coding RNAs (ncRNA), whereas the other two were annotated as miscellaneous RNAs (miscRNA). The regulatory roles of ncRNAs in oxidative stress response and development of cardiac hypertrophy and apoptosis, the biological pathways frequently identified to be associated with myopathies in broilers, were also addressed (Dong et al., 2019; Turunen et al., 2019). In addition, Zhang et al. (2017) identified a set of ncRNAs involved in regulation of intramuscular preadipocyte differentiation in breast muscle of Chinese indigenous chickens, which was similar to the deposition of fat on connective tissue of the WS breasts. Still, specific biological functions of those ncRNAs associated with WS development remain to be elucidated. Overall, the list of the shared DEGs between the comparisons suggests the key genes partly responsible for each WS severity degree.

### Comparison Between Mild and Moderate WS Degrees (C1)

The small numbers of DEGs in C1 relative to the other comparisons suggest slight differences at the cellular level between mild and moderate WS degree. Considering the unique enriched biological pathways altered specifically for C1 (Table 3), the majority of the pathways are linked with phosphatidylinositol phospholipase C (*PLCB*) and *CALM*. The *PLCB*-encoding enzyme catalyzes the hydrolysis of phosphatidylinositol 4,5-bisphosphate into two secondary messengers, inositol triphosphate (IP_3_) and diacylglycerol (DAG), which regulate various transduction cascades, particularly homeostasis of macronutrients and intracellular [Ca^2+^] (Kadamar and Ross, 2013). The *CALM*, the second most differentially expressed gene in C1, plays roles in a vast diversity of cellular functions (Berchtold and Villalobo, 2014). In this study, an increased *PLCB* expression coupled with reduced *CALM* abundance identified in moderate WS compared with those of mild WS appeared to alter metabolisms of macronutrients as well as the biological processes, including circadian clock, dopaminergic synapse, long-term potentiation, gonadotrophin-releasing hormone (GnRH) secretion, melanogenesis, and glucagon signaling (Table 3). Alteration of lipid and amino acid metabolisms could be partly supported by an increase in hepatic aminoadipate aminotransferase (*AADAT*), currently highlighted in the pathway of antibiotic biosynthesis. Upregulation of *AADAT*, reported in liver of fattening pigs, was speculated to promote triglyceride biosynthesis from degraded lysine (Kojima et al., 2018).

**TABLE 3:** The unique Kyoto Encyclopedia of Genes and Genomes (KEGG) pathways highlighted in C1.

	**KEGG pathway**	**KO**	**Gene ID**	**Fold change**
ko01130	Biosynthesis of antibiotics	K00825	*AADAT*	1.21
		K00850	*PFKP*	1.21
		K00869	*MVK*	1.24
		K11787	*GART*	–2.22
ko04713	Circadian entrainment	K02183	*CALM*	–1.31
		K05858	*PLCB*	1.32
		K21945	*PER3*	2.02
ko04728	Dopaminergic synapse	K02183	*CALM*	–1.31
		K04354	*PPP2R2B*	–1.20
		K05858	*PLCB*	1.32
ko04062	Chemokine signaling pathway	K05507	*XCL1*	–1.44
		K05858	*PLCB*	1.32
ko04071	Sphingolipid signaling pathway	K04354	*PPP2R2B*	–1.20
		K05858	*PLCB*	1.32
ko04720	Long-term potentiation	K02183	*CALM*	–1.31
		K05858	*PLCB*	1.32
ko04912	GnRH signaling pathway	K02183	*CALM*	–1.31
		K05858	*PLCB*	1.32
ko04916	Melanogenesis	K02183	*CALM*	–1.31
		K05858	*PLCB*	1.32
ko04922	Glucagon signaling pathway	K02183	*CALM*	–1.31
		K05858	*PLCB*	1.32
ko05142	Chagas disease	K04354	*PPP2R2B*	–1.20
		K05858	*PLCB*	1.32

The association between the dysregulated intracellular [Ca^2+^] and development of moderate WS was emphasized by the 1.4-fold decreased chemokine (C motif) ligand (*XCL1*), encoding lymphotactin as shown in the chemokine signaling pathway (Table 3). Similar to other chemokines, lymphotactin recruits T and natural killer (NK) cells and induces intracellular Ca^2+^ mobilization by binding a specific G-protein-coupled receptor (Volkman et al., 2009).

Among the pathways relevant to dopaminergic synapse, sphingolipid signaling, and Chagas disease, transcript abundance of the beta isoform of regulatory subunit 55 of protein phosphatase 2A (*PPP2R2B*) was decreased 1.2-fold in moderate WS relative to mild samples. Decreased expression of *PPP2R2B* has been linked with the promotion of deregulated oncogene signaling pathways required for survival and proliferation of cancer cells (Tan et al., 2010), suggesting the different cell proliferation rates between mild and moderate WS.

Regarding the circadian entrainment, the changes in the clock gene networks not only associated with the disrupted immunity and metabolic disorders (Turek et al., 2005) but also the structure within the muscle (Harfmann et al., 2015). In this study, apart from *PLCB* and *CALM*, an increased abundance of period circadian regulator 3 (*PER3*) was observed. This gene product plays an important role in adipogenesis as indicated by experiments showing that deletion of *PER3* promoted adipocyte precursor cells in mouse subcutaneous adipose tissue (Aggarwal et al., 2017). Although the expression of *PER3* was upregulated in the C1 comparison in the current study, differential expression of this gene likely affects adipogenesis in these birds and may be partly responsible for the increased fat content within the moderate WS breasts. As the light is the dominant signal for the biological clock-controlled system, the findings imply that the lighting management might partly influence the entanglement of biological cascades, including circadian clock and metabolic functions during the progression of WS from mild to moderate level. Further investigation is required to elucidate such association.

Overall, the current transcriptome profiling consistently suggested the association between the progression of mild to moderate WS and the dysregulated metabolic processes of macronutrients along with the shifts of Ca^2+^ homeostasis. The impacts on amino acids, lipids, and carbohydrate were in accordance with the changes in glycolytic enzymes (Kuttappan et al., 2017) and metabolites from tricarboxylic acid cycle (Boerboom et al., 2018) previously reported in pectoralis major of WS + WB samples, and the differential expression of various genes related to carbohydrate and fatty acid metabolisms identified in WS-affected Cobb 500 broilers (Marchesi et al., 2019).

### Comparison Between Moderate and Severe WS Degrees (C2)

Similar to C1, differential gene expression identified in C2 in which the WS abnormality progressed from moderate to severe levels were related to metabolisms and physiological activities within the abnormal broilers through the signals of phosphatidylinositol, cyclic guanosine monophosphate (cGMP)-dependent protein kinase, and apelin (Figure 4). Consistent with C1, an altered intracellular ion homeostasis, particularly Ca^2+^, was observed in C2. An increased *CALM* abundance in severe WS relative to moderate WS might exert relevant roles in several unique enriched biological pathways, including Ras signaling, cellular senescence, and axon guidance (Table 4). In addition to changes in [Ca^2+^], a difference in [K^+^] between severe and moderate WS might be hypothesized as a consequence of the 1.5-fold increase in transcriptional level of the calcium-activated potassium channel subunit alpha-1 (*KCNMA1*). In salivary secretion pathway, the product of *KCNMA1* functions as a component of the voltage-gated potassium channel, which is activated by either an increased intracellular [Ca^2+^] or changes in membrane electrical potential (Miller, 2000).

**TABLE 4:** The unique Kyoto Encyclopedia of Genes and Genomes (KEGG) pathways highlighted in C2.

	**KEGG Pathway**	**KO**	**Gene ID**	**Fold change**
ko03018	RNA degradation	K00850	*PFKP*	–1.29
		K12572	*PAN3*	–9.57
		K12584	*DCPS*	1.23
ko04014	Ras signaling pathway	K02183	*CALM*	1.25
		K04366	*RAF1*	1.50
		K12362	*RASGRP3*	–1.37
ko04218	Cellular senescence	K02183	*CALM*	1.25
		K04366	*RAF1*	1.50
		K05863	*SLC25A6*	–1.31
ko04360	Axon guidance	K04366	*RAF1*	1.50
		K05112	*EPHB3*	1.52
		K06820	*PLXNA2*	1.30
ko04970	Salivary secretion	K02183	*CALM*	1.25
		K04936	*KCNMA1*	1.53

Unlike C1, the modules containing sequential activities regulating cell proliferation, differentiation, and death, particularly through the pathways of mitogen-activated protein kinase (MAPK) necroptosis, RNA degradation, and cellular senescence (Figure 4), were impacted in C2. In addition, RNA degradation and cellular senescence were among the five unique enriched pathways identified for C2 (Table 4). Considering the complete list of the unique enriched pathways identified in C2 (Table 4), most of them transmit signals in governing cell proliferation and survival. In Ras signaling, Ras functions as a molecular switch for signaling pathways through a cycle of guanidine triphosphates (GTP)- and guanidine diphosphate (GDP)-bound states (Milburn et al., 1990). One of the mechanisms by which GTP-Ras operates includes the recruitment of Raf-1 proteins, encoded by *RAF1*, to form Raf-1 heterodimers, which in turn stimulate various cellular signaling cascades that control cell proliferation, differentiation, and migration (Zenonos and Kyprianou, 2013). As for the axon guidance, its signal cues non-canonically regulate cell proliferation, cell migration, and gene expression (Russell and Bashaw, 2018). It is also interesting that several DEGs in C2 exhibited consistent fold change with the expression required for proliferation and survival of various cancer cell types as addressed in previous studies. For instance, suppressed Ras guanyl-releasing protein 3 (*RASGRP3*) was previously identified in transgenic hypertrophic mouse hearts of which malignant mutations of cardiac myosin regulatory light chain (Huang et al., 2016). In the study of Ji et al. (2011), the abundance of ephrin type-B receptor 3 (*EPHB3*) was positively correlated with severe pathological characteristics, particularly tumor size, in lung cancer cells. Overexpression of *KCNMA1* promoted tumor progression in mouse model (Khaitan and Ningaraj, 2019). Taken together, the underlined molecular shifts when the WS abnormality elevating from moderate to severe lesion was more likely related with dysregulated cell proliferation.

In addition to the potential abnormality in cell proliferation, the transcriptional change specific for C2 exerted more deleterious impact than those observed in C1. The speculation was supported by the two unique enriched pathways, including RNA degradation and cellular senescence. The RNA degradation is required for monitoring RNA species in living cells. Transcriptional changes of a regulatory subunit of poly(A)-nuclease (*PAN3*, decreased by 9.6-fold) and scavenging decapping enzyme (*DCPS*, increased by 1.2-fold) suggest the fluctuations in RNA degradation pathway to a greater extent in severe WS in compared to moderately affected cases. Even small fluctuations are adequate to initiate cell-to-cell variation and interfere with the precision of cell division cycle (Baudrimont et al., 2019). Knockdown of *PFKP* stimulates survival of breast cancer cells (Kim et al., 2017). In addition to *PFKP*, a delayed poly(A) tail degradation associated with reduced PAN3 protein level was observed in HeLa cells subjected to oxidative stress (Yamagishi et al., 2014). The findings underline the more pronounced deviation of cell cycle and accelerated cell proliferation with the increment of WS from moderate to severe level. As for cellular senescence, this pathway is an irreversible cell-cycle arrest induced by a number of endogenous and exogeneous stresses arising from oncogene, DNA damage, and oxidative stress (Zhao et al., 2016). Accumulation of the senescent cells interferes with the functions of stem cells, attenuates muscle regeneration, and promotes an imbalance between bone formation and bone resorption (Baar et al., 2018). Sun et al. (2017) reported an increase in senescent biomarkers in association with isoproterenol-induced pathological cardiac hypertrophy, which also exhibited fibrosis similar to those found in severe WS muscle (Kuttappan et al., 2013; Malila et al., 2019).

The findings in the current microarray study may reflect the uncontrolled proliferation of muscle cells and the disruption of cell cycle within the hypertrophic breast affected with severe WS in comparison to moderate level.

### Comparison Between Mild and Severe WS Degrees (C3)

Microarray data of C3 revealed the greatest numbers of DEGs due potentially to the profound distinctive phenotypic characteristics between the severe and mild groups. Approximately two-thirds of the DEGs in C3 exhibited positive fold changes. Differences, at gene expression level, in cardiovascular system, connective tissue (Table 1), cell cycle, and cellular compromise (Table 2) are highlighted in between severe and mild cases. The enriched biological pathways specific for C3 (Table 5) could reflect the transcriptional alterations of signaling hubs, including phosphatidylinositol 3-kinase (PI3K)/Akt pathway and p53 cascade, and the downstream outputs of such stress-induced adaptive mechanisms.

**TABLE 5:** The unique Kyoto Encyclopedia of Genes and Genomes (KEGG) pathways highlighted in C3.

	**KEGG Pathway**	**KO**	**Gene ID**	**Fold change**
ko04151	PI3K-Akt signaling pathway	K05121	*TEK*	–1.37
		K05631	*MAGI1*	–1.45
		K06482	*ITGA3*	1.48
		K13242	*NOS3*	1.35
		K16340	*PHLPP2*	–1.23
ko04115	p53 signaling pathway	K04398	*casp8*	–1.22
		K10143	*RFWD2*	1.58
		K10146	*CCNG2*	–1.34
ko04210	Apoptosis	K01379	*CTSD*	1.35
		K04398	*casp8*	–1.22
		K04724	*CFLAR*	–1.28
ko04066	HIF-1 signaling pathway	K04515	*CAMK2D*	1.29
		K05121	*TEK*	–1.37
		K13242	*NOS3*	1.35
ko04611	Platelet activation	K13242	*NOS3*	1.35
		K16056	*ORAI1*	1.36
		K19720	*LOC101747382*	1.53
			*LOC107055590*	1.81
			*LOC107055658*	1.39
ko04727	GABAergic synapse	K04851	*CACNA1D*	1.82
		K05175	*GABRA5*	1.30
		K13524	*ABAT*	–1.35
ko04934	Cushing syndrome	K04365	*BRAF*	–1.25
		K04515	*CAMK2D*	1.29
		K04851	*CACNA1D*	1.82
		K16056	*ORAI1*	1.36
ko05160	Hepatitis C virus	K04365	*BRAF*	–1.25
		K04398	*casp8*	–1.22
		K04724	*CFLAR*	–1.28
ko05205	Proteoglycans in cancer	K04365	*BRAF*	–1.25
		K04515	*CAMK2D*	1.29
		K10380	*ANKRD13D*	1.24
ko05016	Huntington disease	K04398	*casp8*	–1.22
		K04648	*DCTN1*	1.56
		K10408	*DNAH3*	–1.25
ko05412	Arrhythmogenic right ventricular	K04851	*CACNA1D*	1.82
	cardiomyopathy	K06482	*ITGA3*	1.48
		K07610	*DES*	1.64

Based on the DEGs enlisted in the PI3K/Akt signaling, it is interesting that several DEGs, including angiopoietin 1-receptor tyrosine kinase (*TEK*), integrin subunit alpha 3 (*ITGA3*), membrane associated guanylate kinase with inverted orientation (*MAGI1*), and nitric oxide synthase 3 (*NOS3*), are found mainly in endothelial cells (Laura et al., 2002; Zhen et al., 2008). The *TEK*-encoding enzyme regulates vascular morphogenesis and homeostasis (Augustin et al., 2009). Targeted deletion of *TEK* contributed to postnatal degeneration of newly formed veins and development of hemangioma-like vascular tufts in mouse retina (Chu et al., 2016). Upregulated *ITGA3* has been linked with a progression of renal failure (Steenhard et al., 2012) and pathological features of liver cancer (Huang et al., 2018). Depletion of *MAGI1* induced upregulation of endoplasmic reticulum (ER) stress-related genes, which in turn signal apoptosis in the endothelial cells (Abe et al., 2019). As demonstrated *in vitro* and *in vivo*, *NOS3* was highly expressed upon the exposure to reactive oxygen species (Zhen et al., 2008). An increase in synthesis of nitric oxide to increase blood flow due to the limited vascularization within WS/WB affected muscle has been speculated (Petracci et al., 2019). As shown in the study of Christov et al. (2007), endothelial cells stimulate myogenic cell (MPCs) growth, whereas the MPCs showed proangiogenic effect upon differentiation. The altered expression of those DEGs supported the hypothesis regarding the imbalanced interplay between the endothelial cells and MPCs within the hypertrophic myofibers in breast muscle of broilers exhibiting severe WS. Still, further studies are necessary to obtain a better understanding underlying specific functions of those DEGs in broiler skeletal muscle.

One of the downstream processes from PI3K-Akt or p53 pathways is the onset of apoptosis with the aim to remove any damaged cells via caspase activity (Chang et al., 2003). Transcript abundance of caspase-8 (*CASP8*), the principal upstream apoptotic caspase effector of death-inducing receptor signaling (Kruidering and Evan, 2000), was decreased by 1.2-fold in severe relative to mild WS samples. A reduced mRNA level of *CASP8* was recently reported in breast tumors collected from the subjects diagnosed with breast cancer (Aghababazadeh et al., 2017). Apart from *CASP8*, the decreases of caspase 8 and Fas-associated death domain-like apoptosis regulator (*CFLAR*) along with the increased cathepsin D (*CTSD*) abundance were identified in severe WS compared to mild samples. The reduced *CFLAR* expression accelerates inflammation, ER stress, and cell death in ischemia-induced mouse brain tissues (Wang et al., 2019), whereas overexpression of *CTSD* was linked with various diseases, e.g., type 2 diabetes (Liu et al., 2017), acute coronary syndromes (Gonçalves et al., 2016), and malignant epithelial tumor (Zhang et al., 2018). Additionally, the extensive alteration of apoptosis in severe WS is emphasized by the increased abundance of dynactin subunit 1 (*DCTN1*) coupled with decreased axonemal heavy chain 3 of dynein (*DNAH3*), highlighted in Huntington disease pathway (Table 5). Differential expression of those two genes inhibits apoptotic membrane trafficking during apoptosis (Lane et al., 2001). Overall, the modulated programmed cell death could be anticipated in severe WS.

The importance of altered intracellular [Ca^2+^] in the development of WS was again underlined by the DEGs identified in C3. The Ca^2+^-/calmodulin-dependent protein kinase type II delta chain (*CAMK2D*) is part of a complex that regulates ER-mediated Ca^2+^ influx in either skeletal or cardiac muscles. The Ca^2+^ voltage-gated channel subunit alpha 1D (*CACNA1D*) is specifically part of the channel responsible for conferring low-voltage activation and slowly inactivating Ca^2+^ currents. The Ca^2+^ release-activated calcium channel protein 1 (*ORAI1*), a Ca^2+^-selective channel particularly in T cells, mediates Ca^2+^ influx following the depletion of intracellular Ca^2+^ stores (Gwozdz et al., 2012). Overexpression of *CAMK2D* and *CACNA1D* induces cardiac enlargement, with mild fibrosis resembling the severe WS lesion, in transgenic mice (Zhang et al., 2003). In addition, a decreased expression of the aminobutyrate aminotransferase (*ABAT*), highlighted in GABAergic signaling, is associated with increased intracellular [Ca^2+^], leading to an increased Ca^2+^-sensing nuclear translocation associated with aggressive breast cancer (Chen et al., 2019). The changes in transcription of those DEGs likely lead to the excessive Ca^2+^ influx in severe WS samples (Kuttappan et al., 2013; Zambonelli et al., 2016; Marchesi et al., 2019).

Increased abundances of *LOC101747382*, *LOC107055590*, and *LOC107055658*, encoding type I collagen, coupled with upregulated *NOS3* and *ORAI1*, were highlighted under the pathway of platelet activation. The pathway is responsible primarily for coagulation cascades upon the interaction between platelet membrane glycoproteins and exposed collagens and secondarily for intercellular communication that mediates inflammatory and immunomodulatory activities, particularly with the triggers from increasing intracellular [Ca^2+^] and extracellular matrix (Yun et al., 2016). Generally, the ratio between types 1 and 3 determines mechanical properties of collagen matrix, hence the tissues. An increase in proportion of collagen type 1 has been demonstrated in healing wounds, hypertensive arteries, and fibrotic tissues (Friedman et al., 1993), which was in accordance with fibrotic characteristics of severe WS previously described (Trocino et al., 2015).

Regarding the arrhythmogenic right ventricular cardiomyopathy, the pathological characteristics of this heart muscle disease includes progressive myocyte loss and fibrofatty replacement, which were resembled to the histological lesions of severe WS. Under this enriched pathway, increased transcript abundance of desmin (*DES*) was significant in severe WS in comparison to that of mild WS. This observation was consistent with the previous report on the induced DES at the protein levels in WS/WB breast muscle samples compared with nondefective ones (Zambonelli et al., 2016; Kuttappan et al., 2017; Soglia et al., 2020). Based on the functions of desmin in structural organization of myofibers during myogenesis, the greater *DES* in severe WS suggested the occurrence of extensive muscle regeneration within the severe WS in comparison with those of found in mild WS samples.

The majority of the DEGs enlisted in the unique pathways of C3 were also associated with growth and proliferation of cancer cells as observed in C1 and C2. Apart from those DEGs mentioned above, transcript abundance of plekstrin homology domain leucine-rich repeat-containing protein phosphatase 2 (*PHLPP2*), a negative regulator of PI3K/Akt pathway, was decreased by 1.2-fold. A loss of *PHLPP2* is associated with progression of pancreatic (Smith et al., 2016) and prostatic cancer (Nowak et al., 2019). Differential expressions of cyclin G (*CCNG*) and increased RING finger and WD repeat domain protein 2 (*RFWD2*), the two proteins involved in negative autoregulatory feedback loops of the p53 response observed in this study, were concomitant with the modified transcriptional direction addressed in gastric cancer tissues and in ovarian and breast cancer tissues, respectively (Dornan et al., 2004; Gao et al., 2018). Decreased expression of the gene encoding B-Raf (*BRAF*) by 1.2-fold in severe compared to mild WS might imply the impaired proliferative arrest in the severe WS.

HIF-1 signaling is also highlighted in C3. The pathway, consistently identified in the WS/WB myopathies (Malila et al., 2019; Marchesi et al., 2019; Pampouille et al., 2019), stimulates an adaptive mechanism via an activation of transcription factor HIF-1 when cells sense low oxygen availability. Despite no difference in expression of HIF-1, the shifts of *TEK* and *NOS3* expression suggested modified angiogenesis and antioxidant activities under hypoxia within the severe WS compared with those in mild WS samples. The current results correspond well with our previous report (Malila et al., 2019) in which an increased *HIF1A* was only detected when absolute transcription level of WS and WS + WB muscle were compared with that of nondefective ones; however, no change was observed among the defective samples. This might explain the absence of *HIF1A* in the DEGs obtained in this report.

Collectively, the changes in gene expression patterns combined with the unique biological pathways in C3 were in the same direction related to development and proliferation of tumor and cancer in several tissue types. The molecular resemblance underlined the dysregulation of damaged cells as well as the aberrant cell proliferation potentially during muscle regeneration within the muscle exhibiting severe WS myopathy.

### Altered Biological Pathways in Common Among All Comparisons

Most of the overlapping enriched pathways comprised the same set of the DEGs discussed in each comparison, suggesting the importance of those DEGs in the progression of WS condition. The shared altered pathways emphasize the impact of dysregulated intracellular ion homeostasis and modulated cellular cascades that remove damaged cells in relevance with WS abnormality. In this study, transcript abundances of *PLCB*, *CACNA1D*, *CAMK2*, and *ORAI1* gradually increased during WS progression. The findings were consistent with previous studies of Zambonelli et al. (2016) and Marchesi et al. (2019) identifying upregulation of those genes in WS-related muscle compared with those of nondefective ones. Increases in expression of those genes have been related with elevated intracellular [Ca^2+^]. As Ca^2+^ is one of the crucial secondary messengers associated with phosphoinositol system, [Ca^2+^] disturbance may affect both the molecular functions and the viability of the cell. Elevated intracellular [Ca^2+^] modulates apoptosis and protein degradation, compromises mitochondrial function, and interferes with cell membrane integrity (Florea et al., 2005; Mutryn et al., 2015; Petracci et al., 2015). This could explain the extensive muscle protein degradation observed in WS samples collected from Cobb 500 broilers in the previous report of Vignale et al. (2017). Likewise, *NOS3* was increased as WS severity increased. This result supported the speculation of Petracci et al. (2019), suggesting the elevated hypoxic condition as WS severity elevated. In addition, the gene encoding solute carrier family 25 (*SLC25A4*) slightly decreased as the myopathy progressed. Expression of this gene promoted oxidative stress and apoptosis (Lena et al., 2010). A progressive decrease in *PHKB* was detected, which agreed well with the previous findings of Marchesi et al. (2019), suggesting an unusual muscle contraction process within the affected muscle.

Consistent with the observation in C2 and C3, the numbers of the DEGs obtained in the overlapped pathways were changed in the direction that facilitated proliferation and progression of cancer cells and tumors (Figure 6). Apart from the DEGs discussed earlier, the DNA mismatch repair protein, encoded by *MSH6*, gradually decreased while the WS severity increased. Previously, suppressed *MSH6* was detected under an experimental hypoxic condition (Koshiji et al., 2005). The shifts of glutathione (*GST*)-encoding gene among the WS severity levels, corresponding with our recent ddPCR results (Malila et al., 2019), implied responsive mechanism of the muscle cells against increased oxidative stress. In addition to impaired apoptosis, ubiquitination-mediated proteolysis (Supplementary Figure S1) was disrupted. An accumulation of the unrepaired oxidative damaged biomolecules could induce a cascade of molecular events that ultimately contribute to promotion of cancer or tumor growth (Conde-Pérezprina et al., 2012). The current microarray results strengthen the case for oxidative stress-induced damage within the WS breast that induce a cascade of molecular events contributing to aberrant cell proliferation.

Although the actual etiology regarding development of WS abnormality remains under investigation, the current transcriptome analyses ascertained an association between disturbances of calcium homeostasis during progression of WS severity. In complement to the previous report, our results underlined the major shifts of biological processes while the abnormality progressed. When WS abnormality advanced from mild to moderate WS, the microarray data revealed transcriptional changes of the key upstream mediators of Ca^2+^. The impacts of imbalanced intracellular [Ca^2+^] coupled with alterations of the other secondary messengers may exert an overall metabolic shift and inflammation. As the WS myopathy progresses to the severe degree, a more profound perturbation of intracellular [Ca^2+^] along with oxidative stress could be anticipated. One of the major origins of the oxidative stress might potentially be the limited capillarization. The differences in metabolisms of macronutrients and small biochemical molecules might be shadowed by the extensive results of impaired cell-cycle arrest and cell proliferation. With the unrepaired DNA mismatches along with modulated programmed cell death, accumulation of abnormal genomic materials and intermediate metabolites could trigger dysregulation of physiological functions and excessive cell proliferation. The aberrant muscle cells underwent necrosis as supported by the previous histological reports (Kuttappan et al., 2013; Malila et al., 2018; Marchesi et al., 2019) and ultimately developed severe WS characteristics.

In conclusion, the current transcriptome analysis strengthens the association between development of WS abnormality in commercial broilers and aberrant molecular mechanisms responsible for controlling ion homeostasis and muscle repair process. The transcriptional alteration, when WS progressed from mild to moderate, also influenced metabolisms of macronutrients and small biomolecules. In the development of severe WS lesion, besides the potential leak of the ions, the changes in expression of several genes resemble those found in highly proliferating cells. In addition to the link with an intensive selection for fast-growing and enlarged muscle mass, this study has shown an alteration of the circadian clock system at transcriptional level. The current findings offer evidence that both the intensive, selective breeding and overall management of the modern broiler may play roles in development and severe progression of this myopathy. Optimizing rather than maximizing growth performance of broilers should be prioritized to ensure security of this inexpensive protein source and the sustainability of poultry industry.

## Data Availability Statement

The datasets generated for this study can be found in the NCBI GEO Series GSE107362 (https://www.ncbi.nlm.nih.gov/geo/query/acc.cgi?acc=GSE107362), Platform GPL24307 (https://www.ncbi.nlm.nih.gov/geo/query/acc.cgi?acc=GPL24307).

## Ethics Statement

All samples were purchased in the form of whole carcasses from the commercial processing plant. Neither experimental treatments nor scientific procedures were subjected to the living animals. Thereby, according to BIOTEC Institutional Animal Care and Use Committee, the ethical approval was not exempted.

## Author Contributions

YM, WR, and WV conceived and designed the experiments as well as acquired the funding. YM, TU, YS, SA, and KT performed the experiments and analyzed the data. YM, TU, WR, FS, and GS drafted the manuscript. All authors read and approved the final manuscript.

## Conflict of Interest

The authors declare that the research was conducted in the absence of any commercial or financial relationships that could be construed as a potential conflict of interest.

## Footnotes

1 https://www.ncbi.nlm.nih.gov/tools/primer-blast/

## References

[B1] Abe J. I. Ko K. A. Kotla S. Wang Y. Paez-Mayorga J. Shin I. J. (2019). MAGI1 as a link between endothelial activation and ER stress drives atherosclerosis. *JCI Insight.* 4:e125570. 10.1172/jci.insight.125570 30944250 PMC6483653

[B2] Aggarwal A. Costa M. J. Rivero-Gutiérrez B. Ji L. Morgan S. L. Feldman B. J. (2017). The circadian clock regulates adipogenesis by a Per3 crosstalk pathway to Klf15. *Cell Rep.* 21 2367–2375. 10.1016/j.celrep.2017.11.004 29186676 PMC5728416

[B3] Aghababazadeh M. Dorraki N. Javan F. A. Fattahi A. S. Gharib M. Pasdar A. (2017). Downregulation of caspase 8 in a group of Iranian breast cancer patients - a pilot study. *J. Egypt. Natl. Canc. Inst.* 29 191–195. 10.1016/j.jnci.2017.10.001 29233452

[B4] Augustin H. G. Koh G. Y. Thurston G. Alitalo K. (2009). Control of vascular morphogenesis and homeostasis through the angiopoietin-Tie system. *Nat. Rev. Mol. Cell Biol.* 10 165–177. 10.1038/nrm2639 19234476

[B5] Baar M. P. Perdiguero E. Muñoz-Cánoves P. de Keizer P. L. (2018). Musculoskeletal senescence: a moving target ready to be eliminated. *Curr. Opin. Pharmacol.* 40 147–155. 10.1016/j.coph.2018.05.007 29883814

[B6] Baudrimont A. Jaquet V. Wallerich S. Voegeli S. Becskei A. (2019). Contribution of RNA degradation to intrinsic and extrinsic noise in gene expression. *Cell Rep.* 26 3752–3761. 10.1016/j.celrep.2019.03.001 30917326

[B7] Berchtold M. W. Villalobo A. (2014). The many faces of calmodulin in cell proliferation, programmed cell death, autophagy, and cancer. *BBA Mol. Cell. Res.* 1843 398–435. 10.1016/j.bbamcr.2013.10.021 24188867

[B8] Boerboom G. van Kempen T. Navarro-Villa A. Pèrez-Bonilla A. (2018). Unraveling the cause of white striping in broilers using metabolomics. *Poult. Sci.* 97:3977. 10.3382/ps/pey266 29931266 PMC6162359

[B9] Bolstad B. (2017). *PreprocessCore**: A Collection Of Pre-Processing Functions. R Package Version 1.40.0.* Available at: https://github.com/bmbolstad/preprocessCore (accessed July 23, 2018).

[B10] Camarillo C. Swerdel M. Hart R. P. (2011). Comparison of microarray and quantitative real-time PCR methods for measuring MicroRNA levels in MSC cultures. *Methods Mol. Biol.* 698 419–429. 10.1007/978-1-60761-999-4_30 21431535 PMC4442613

[B11] Chan K. K. M. Seetharaman A. Bagg R. Selman G. Zhang Y. Kim J. (2014). EVA-1 functions as an UNC-40 Co-receptor to enhance attraction to the MADD-4 guidance cue in *Caenorhabditis elegans*. *PLoS Genet.* 10:e1004521. 10.1371/journal.pgen.1004521 25122090 PMC4133157

[B12] Chang F. Lee J. T. Navolanic P. M. Steelman L. S. Shelton J. G. Blalock W. L. (2003). Involvement of PI3K/Akt pathway in cell cycle progression, apoptosis, and neoplastic transformation: a target for cancer chemotherapy. *Leukemia* 17 590–603. 10.1038/sj.leu.2402824 12646949

[B13] Chen X. Cao Q. Liao R. Wu X. Xun S. Huang J. (2019). Loss of ABAT-mediated GABAergic system promotes basal-like breast cancer progression by activating Ca2+-NFAT1 axis. *Theranostics* 9 34–47. 10.7150/thno.29407 30662552 PMC6332792

[B14] Christov C. Chrétien F. Abou-Khalil R. Bassez G. Vallet G. Authier F. J. (2007). Muscle satellite cells and endothelial cells: close neighbors and privileged partners. *Mol. Biol. Cell.* 18 1397–1409. 10.1091/mbc.e06-08-0693 17287398 PMC1838982

[B15] Chu M. Li T. Shen B. Cao X. Zhong H. Zhang L. (2016). Angiopoietin receptor Tie2 is required for vein specification and maintenance via regulating COUP-TFII. *eLife* 5:e21032. 10.7554/eLife.21032 28005008 PMC5218530

[B16] Conde-Pérezprina J. C. León-Galván M. Á Konigsberg M. (2012). DNA mismatch repair system: repercussions in cellular homeostasis and relationship with aging. *Oxid. Med. Cell. Longev.* 2012:728430. 10.1155/2012/728430 23213348 PMC3504481

[B17] Datta K. Fuentes J. L. Maddok J. R. (2005). The yeast GTPase Mtg2p is required for mitochondrial translation and partially suppresses an rRNA methyltransferase mutant, mrm2. *Mol. Biol. Cell.* 16 954–963. 10.1091/mbc.e04-07-0622 15591131 PMC545925

[B18] De Jong A. Buchili R. Robbins D. (2002). Characterization of sperm protein 17 in human somatic and neoplastic tissue. *Cancer Lett.* 186 201–209. 10.1016/s0304-3835(02)00350-6 12213290

[B19] Dong Y. Xu W. Liu C. Liu P. Li P. Wang K. (2019). Reactive oxygen species related noncoding RNAs as regulators of cardiovascular diseases. *Int. J. Biol. Sci.* 15 680–687. 10.7150/ijbs.30464 30745854 PMC6367576

[B20] Dornan D. Bheddah S. Newton K. Ince W. Frantz G. D. Dowd P. (2004). COP1, the negative regulator of p53, is overexpressed in breast and ovarian adenocarcinomas. *Cancer Res.* 64 7226–7230. 10.1158/0008-5472.CAN-04-2601 15492238

[B21] Florea A. M. Yamoah E. N. Dopp E. (2005). Intracellular calcium disturbances induced by arsenic and its methylated derivatives in relation to genomic damage and apoptosis induction. *Environ. Health Perspect.* 113 659–664. 10.1289/ehp.7634 15929885 PMC1257587

[B22] Friedman D. W. Boyd C. D. Norton P. Greco R. S. Boyarsky A. H. Mackenzie J. W. (1993). Increases in type III collagen gene expression and protein synthesis in patients with inguinal hernias. *Ann. Surg.* 218 754–760. 10.1097/00000658-199312000-00009 7710461 PMC1243071

[B23] Gao J. Zhao C. Liu Q. Hou X. Li S. Xing X. (2018). Cyclin G2 suppresses Wnt/β-catenin signaling and inhibits gastric cancer cell growth and migration through Dapper1. *J Exp. Clin. Cancer Res.* 37:317. 10.1186/s13046-018-0973-2 30547803 PMC6295076

[B24] Gonçalves I. Hultman K. Dunér P. Edsfeldt A. Hedblad B. Fredrikson G. N. (2016). High levels of cathepsin D and cystatin B are associated with increased risk of coronary events. *Open Heart* 3:e000353. 10.1136/openhrt-2015-000353 26848396 PMC4731836

[B25] Gwozdz T. Dutko-Gwozdz J. Schafer C. Bolotina V. M. (2012). Overexpression of Orai1 and STIM1 proteins alters regulation of store-operated Ca2+ entry by endogenous mediators. *J. Biol. Chem.* 287 22865–22872. 10.1074/jbc.M112.356626 22549787 PMC3391152

[B26] Hamilton S. L. Serysheva I. Strasburg G. M. (2000). Calmodulin and excitation-contraction coupling. *News Physiol. Sci.* 15 281–284. 10.1152/physiologyonline.2000.15.6.281 11390927

[B27] Harfmann B. D. Schroder E. A. Esser K. A. (2015). Cirdadian rhythms, the molecular clock, and skeletal muscle. *J. Biol. Rhythms* 30 84–94. 10.1177/0748730414561638 25512305 PMC4470613

[B28] Huang W. Kazmierczak K. Zhou Z. Aguiar-Pulido V. Narasimhan G. Szczesna-Cordarya D. (2016). Gene expression patterns in transgenic mouse models of hypertrophic cardiomyopathy caused by mutations in myosin regulatory light chain. *Arch. Biochem. Biophys.* 601 121–132. 10.1016/j.abb.2016.02.022 26906074 PMC5370580

[B29] Huang Y. Kong Y. Zhang L. He T. Zhou X. Yan Y. (2018). High expression of ITGA3 promotes proliferation and cell cycle progression and indicates poor prognosis in intrahepatic cholangiocarcinoma. *BioMed. Res. Int.* 2018:2352139. 10.1155/2018/2352139 29511671 PMC5817212

[B30] Ji X. D. Li G. Feng Y. X. Zhao J. S. Li J. J. Sun Z. J. (2011). EphB3 is overexpressed in non-small-cell lung cancer and promotes tumor metastasis by enhancing cell survival and migration. *Cancer Res.* 71 1156–1166. 10.1158/0008-5472.CAN-10-0717 21266352

[B31] Kadamar G. Ross E. M. (2013). Mammalian phospholipase C. *Annu. Rev. Physiol.* 75 127–154. 10.1146/annurev-physiol-030212-183750 23140367

[B32] Kanehisa M. Goto S. Sato Y. Furumichi M. Tanabe M. (2012). KEGG for integration and interpretation of large-scale molecular data sets. *Nucleic Acids Res.* 40 D109–D114. 10.1093/nar/gkr988 22080510 PMC3245020

[B33] Khaitan D. Ningaraj N. (2019). Evidence of calcium-activated potassium channel subunit alpha-1 as a key promoter of glioma growth and tumorigenicity. *Glioma* 2 46–54. 10.4103/glioma.glioma_44_18

[B34] Kim N. H. Cha Y. H. Lee J. Lee S. H. Yang J. H. Yun J. S. (2017). Snail reprograms glucose metabolism by repressing phosphofructokinase PFKP allowing cancer cell survival under metabolic stress. *Nat. Commun.* 8:14374. 10.1038/ncomms14374 28176759 PMC5309788

[B35] Koivunen P. Tiainen P. Hyvärinen J. Williams K. E. Sormunen R. Klaus S. J. (2007). An endoplasmic reticulum transmembrane prolyl 4-hydroxylase is induced by hypoxia and acts on hypoxia-inducible factor α. *J. Biol. Chem.* 282 30544–30552. 10.1074/jbc.M704988200 17726031

[B36] Kojima M. Nakajima I. Arakawa A. Mikawa S. Matsumoto T. Uenishi H. (2018). Differences in gene expression profiles for subcutaneous adipose, liver, and skeletal muscle tissues between Meishan and Landrace pigs with different backfat thicknesses. *PLoS One* 13:e0204135. 10.1371/journal.pone.0204135 30240433 PMC6150482

[B37] Koshiji M. To K. K. Hammer S. Kumamoto K. Harris A. L. Modrich P. (2005). HIF-1α induces genetic instability by transcriptionally downregulating MutSα Expression. *Mol. Cell.* 17 793–803. 10.1016/j.molcel.2005.02.015 15780936

[B38] Kruidering M. Evan G. I. (2000). Caspase-8 in apoptosis: the beginning of “the end”? *IUBMB Life* 50 85–90. 10.1080/713803693 11185963

[B39] Kuttappan V. A. Bottje W. Ramnathan R. Hartson S. D. Coon C. N. Kong B. W. (2017). Proteomic analysis reveals changes in carbohydrate and protein metabolism associated with broiler breast myopathy. *Poult. Sci.* 96 2992–2999. 10.3382/ps/pex069 28499042

[B40] Kuttappan V. A. Hargis B. M. Owens C. M. (2016). White striping and woody breast myopathies in the modern poultry industry: a review. *Poult. Sci.* 95 2724–2733. 10.3382/ps/pew216 27450434

[B41] Kuttappan V. A. Lee Y. S. Erf G. F. Meullenet J. F. McKee S. R. Owens C. M. (2012). Consumer acceptance of visual appearance of broiler breast meat with varying degrees of white striping. *Poult. Sci.* 91 1240–1247. 10.3382/ps.2011-01947 22499884

[B42] Kuttappan V. A. Shivaprasad H. L. Shaw D. P. Valentine B. A. Hargis B. M. Clark F. D. (2013). Pathological changes associated with white striping in broiler breast muscles. *Poult. Sci.* 92 331–338. 10.3382/ps.2012-02646 23300297

[B43] Lane J. D. Vergnolle M. A. Woodman P. G. Allan V. J. (2001). Apoptotic cleavage of cytoplasmic dynein intermediate chain and p150(Glued) stops dynein-dependent membrane motility. *J. Cell Biol.* 153 1415–1426. 10.1083/jcb.153.7.1415 11425872 PMC2150727

[B44] Laura R. P. Ross S. Koeppen H. Lasky L. A. (2002). MAGI-1: a widely expressed, alternatively spliced tight junction protein. *Exp. Cell Res.* 275 155–170. 10.1006/excr.2002.5475 11969287

[B45] Leek J. McShane B. B. Gelman A. Colquhoun D. Nuijten M. B. Goodman S. N. (2017). Five ways to fix statistics. *Nature* 551 557–559. 10.1038/d41586-017-07522-z 29189798

[B46] Lena A. Rechichi M. Salvetti A. Vecchio D. Evangelista M. Rainaldi G. (2010). The silencing of adenine nucleotide translocase isoform 1induces oxidative stress and programmed cell death in ADF human glioblastoma cells. *FEBS J.* 277 2853–2867. 10.1111/j.1742-4658.2010.07702.x 20528917

[B47] Liu L. Chen B. Zhang X. Tan L. Wang D. W. (2017). Increased cathepsin D correlates with clinical parameters in newly diagnosed type 2 diabetes. *Dis. Markers* 2017:5286408. 10.1155/2017/5286408 29375176 PMC5742441

[B48] Livak K. J. Schmittgen T. D. (2001). Analysis of relative gene expression data using real-time quantitative PCR and the 2(-Delta Delta C(T) Method. *Methods* 25 402–408. 10.1006/meth.2001.1262 11846609

[B49] Malila Y. Srimarut Y. U-chupaj J. Strasburg G. Visessanguan W. (2015). Monitoring of chicken RNA integrity as a function of prolonged postmortem duration. *Asian Austr. J. Anim. Sci.* 28 1649–1656. 10.5713/ajas.15.0167 26580287 PMC4647106

[B50] Malila Y. Thanatsang K. Arayamethakorn S. Uengwetwanit T. Srimarut Y. Petracci M. (2019). Absolute expressions of hypoxia-inducible factor-1 alpha (HIF1A) transcript and the associated genes in chicken skeletal muscle with white striping and wooden breast myopathies. *PLoS One* 14:e0220904. 10.1371/journal.pone.0220904 31393948 PMC6687142

[B51] Malila Y. U-chupaj J. Srimarut Y. Chaiwiwattrakul P. Uengwetwanit T. Arayamethakorn S. (2018). Monitoring of white striping and wooden breast cases and impacts on quality of breast meat collected from commercial broilers (Gallus gallus). *Asian Austr. J. Anim. Sci.* 31 1807–1817. 10.5713/ajas.18.0355 30145875 PMC6212750

[B52] Marchesi J. A. P. Ibelli A. M. G. Peixoto J. O. Cantão M. E. Pandolfi J. R. C. Marciano C. M. M. (2019). Whole transcriptome analysis of the pectoralis major muscle reveals molecular mechanisms involved with white striping in broiler chickens. *Poult. Sci.* 98 590–601. 10.3382/ps/pey429 30239913

[B53] Milburn M. Tong L. deVos A. Brunger A. Yamaizumi Z. Nishimura S. (1990). Molecular switch for signal transduction: structural differences between active and inactive forms of protooncogenic ras proteins. *Science* 247 939–945. 10.1126/science.2406906 2406906

[B54] Miller C. (2000). An overview of the potassium channel family. *Genome Biol.* 1:reviews0004.1. 10.1186/gb-2000-1-4-reviews0004 11178249 PMC138870

[B55] Müller C. Schillert A. Röthemeier C. Trégouët D. A. Proust C. Binder H. (2016). Removing batch effects from longitudinal gene expression - quantile normalization plus ComBat as best approach for microarray transcriptome data. *PLoS One* 11:e0156594. 10.1371/journal.pone.0156594 27272489 PMC4896498

[B56] Mutryn M. F. Brannick E. M. Fu W. Lee W. R. Abasht B. (2015). Characterization of a novel chicken muscle disorder through differential gene expression and pathway analysis using RNA-sequencing. *BMC Genomics* 16:399. 10.1186/s12864-015-1623-0 25994290 PMC4438523

[B57] Nowak D. G. Katsenelson K. C. Watrud K. E. Chen M. Mathew G. D’Andrea V. D. (2019). The PHLPP2 phosphatase is a druggable driver of prostate cancer progression. *J. Cell Biol.* 218 1943–1957. 10.1083/jcb.201902048 31092557 PMC6548123

[B58] Pampouille E. Hennequet-Antier C. Praud C. Juanchich A. Brionne A. Godet E. (2019). Differential expression and co- expression gene network analyses reveal molecular mechanisms and candidate biomarkers involved in breast muscle myopathies in chicken. *Sci. Rep.* 9:14905. 10.1038/s41598-019-51521-1 31624339 PMC6797748

[B59] Petracci M. Cavini C. (2012). Muscle growth and poultry meat quality issues. *Nutrients* 4 1–12. 10.3390/nu4010001 22347614 PMC3277097

[B60] Petracci M. Mudalal S. Babini E. Cavani C. (2014). Effect of white striping on chemical composition and nutritional value of chicken breast meat. *Ital. J. Anim. Sci.* 13 178–183. 10.4081/ijas.2014.3138

[B61] Petracci M. Mudalal S. Soglia F. Cavani C. (2015). Meat quality in fast-growing broiler chickens. *Worlds Poult. Sci. J.* 71 363–374. 10.1017/S0043933915000367

[B62] Petracci M. Sirri F. Mazzoni M. Meluzzi A. (2013). Comparison of breast muscle traits and meat quality characteristics in 2 commercial chicken hybrids. *Poult. Sci.* 92 2438–2447. 10.3382/ps.2013-03087 23960128

[B63] Petracci M. Soglia F. Madruga M. Carvalho L. Ida E. Estévez M. (2019). Wooden-breast, white striping, and spaghetti meat: causes, consequences and consumer perception of emerging broiler meat abnormalities. *Compr. Rev. Food Sci.* 18 565–583. 10.1111/1541-4337.12431 33336940

[B64] Roll-Mecak A. Vale R. D. (2008). Structural basis of microtuble severing by the hereditary spastic paraplegia protein spastin. *Nature* 451 363–367. 10.1038/nature06482 18202664 PMC2882799

[B65] Russell S. A. Bashaw G. J. (2018). Axon guidance pathways and the control of gene expression. *Dev. Dyn.* 247 571–580. 10.1002/dvdy.24609 29226467 PMC6167058

[B66] Smith A. J. Wen Y. A. Stevens P. D. Liu J. Wang C. Gao T. (2016). PHLPP negatively regulates cell motility through inhibition of Akt activity and integrin expression in pancreatic cancer cells. *Oncotarget* 7 7801–7815. 10.18632/oncotarget.6848 26760962 PMC4884955

[B67] Soglia F. Mazzoni M. Zappaterra M. Di Nunzio M. Babini E. Bordini M. (2020). Distribution and expression of vimentin and desmin in broiler pectoralis major affected by the growth-related muscular abnormalities. *Front. Physiol.* 10:1581. 10.3389/fphys.2019.01581 32009982 PMC6978684

[B68] Steenhard B. M. Vanacore R. Friedman D. Zelenchuk A. Stroganova L. Isom K. (2012). Upregulated expression of integrin α1 in mesangial cells and integrin α3 and vimentin in podocytes of Col4a3-null (Alport) mice. *PLoS One* 7:e50745. 10.1371/journal.pone.0050745 23236390 PMC3517557

[B69] Sun R. Zhu B. Xiong K. Sun Y. Shi D. Chen L. (2017). Senescence as a novel mechanism involved in β-adrenergic receptor mediated cardiac hypertrophy. *PLoS One* 12:e0182668. 10.1371/journal.pone.0182668 28783759 PMC5544424

[B70] Tan J. Lee P. L. Li Z. Jiang X. Lim Y. C. Hooi S. C. (2010). B55β-associated PP2A complex controls PDK1-directed Myc signaling and modulates rapamycin sensitivity in colorectal cancer. *Cancer Cell* 18 459–471. 10.1016/j.ccr.2010.10.021 21075311

[B71] Trocino A. Piccirillo A. Birolo M. Radaelli G. Bertotto D. Filiou E. (2015). Effect of genotype, gender and feed restriction on growth, meat quality and the occurrence of white striping and wooden breast in broiler chickens. *Poult. Sci.* 94 2996–3004. 10.3382/ps/pev296 26475069

[B72] Turek F. W. Joshu C. Kohsaka A. Lin E. Ivanova G. (2005). Obesity and metabolic syndrome in circadian clock mutant mice. *Science* 308 1043–1045. 10.1126/science.1108750 15845877 PMC3764501

[B73] Turunen T. A. Roberts T. C. Laitinen P. Väänänen M. A. Korhonen P. Malm T. (2019). Changes in nuclear and cytoplasmic microRNA distribution in response to hypoxic stress. *Sci. Rep.* 9:10332. 10.1038/s41598-019-46841-1 31316122 PMC6637125

[B74] Vignale K. Caldas J. V. England J. A. Boonsinchai N. Magnuson A. Pollock E. D. (2017). Effect of white striping myopathy on breast muscle (Pectoralis major) protein turnover and gene expression in broilers. *Poult. Sci.* 96 886–893. 10.3382/ps/pew315 27665016

[B75] Volkman B. F. Liu T. Y. Peterson F. C. (2009). Lymphotactin structural dynamics. *Methods Enzymol.* 461 51–70. 10.1016/S0076-6879(09)05403-2 19480914 PMC3686570

[B76] Wang X. Zhao J. Guo H. Fan Q. (2019). CFLAR is a critical regulator of cerebral ischaemia-reperfusion injury through regulating inflammation and endoplasmic reticulum (ER) stress. *Biomed. Pharmacother.* 117:109155. 10.1016/j.biopha.2019.109155 31387178

[B77] Yamagishi R. Hosoda N. Hoshino S. (2014). Arsenite inhibits mRNA deadenylation through proteolytic degradation of Tob and Pan3. *Biochem. Biophys. Res. Commun.* 455 323–331. 10.1016/j.bbrc.2014.11.015 25446091

[B78] Yun S. H. Sim E. H. Goh R. Y. Park J. I. Han J. Y. (2016). Platelet activation: the mechanisms and potential biomarkers. *Biomed. Res. Int.* 2016:9060143. 10.1155/2016/9060143 27403440 PMC4925965

[B79] Zambonelli P. Zappaterra M. Soglia F. Petracci M. Sirri F. Cavani C. (2016). Detection of differentially expressed genes in broiler pectoralis major muscle affected by white striping - wooden breast myopathies. *Poult. Sci.* 95 2771–2785. 10.3382/ps/pew268 27591279

[B80] Zenonos K. Kyprianou K. (2013). RAS signaling pathways, mutations and their role in colorectal cancer. *World J. Gastrointest. Oncol.* 5 97–101. 10.4251/wjgo.v5.i5.97 23799159 PMC3682174

[B81] Zhang M. Wu J. S. Yang X. Pang X. Li L. Wang S. S. (2018). Overexpression cathepsin D contributes to perineural invasion of salivary adenoid cystic carcinoma. *Front. Oncol.* 8:492. 10.3389/fonc.2018.00492 30430081 PMC6220369

[B82] Zhang T. Maier L. S. Dalton N. D. Miyamoto S. Ross J. Jr. Bers D. M. (2003). The δC isoform of CaMKII is activated in cardiac hypertrophy and induces dilated cardiomyopathy and heart failure. *Circ. Res.* 92 912–919. 10.1161/01.RES.0000069686.31472.C5 12676814

[B83] Zhang T. Zhang X. Han K. Zhang G. Wang J. Xie K. (2017). Analysis of long noncoding RNA and mRNA using RNA sequencing during the differentiation of intramuscular preadipocytes in chicken. *PLoS One* 12:e0172389 10.1371/journal.pone.01172389 PMC5310915 28199418

[B84] Zhao M. Chen L. Qu H. (2016). CSGene: a literature-based database for cell senescence genes and its application to identify critical cell aging pathways and associated diseases. *Cell Death Dis.* 7:e2053. 10.1038/cddis.2015.414 26775705 PMC4816187

[B85] Zhen J. Lu H. Wang X. Q. Vaziri N. D. Zhou X. J. (2008). Upregulation of endothelial and inducible nitric oxide synthase expression by reactive oxygen species. *Am. J. Hypertens.* 21 28–34. 10.1038/ajh.2007.14 18091741

