# Supplementary material for: Transcriptional Profiles of Skeletal Muscle Associated With Increasing Severity of White Striping in Commercial Broilers
Source: Front Physiol. 2020 Jun 16;11:580. doi: 10.3389/fphys.2020.00580 (PMC7308426; doi:10.3389/fphys.2020.00580)
Supplement: Supplementary file 4 [file Data_Sheet_1.zip › supplementary figures.pptx]

## Slide 1
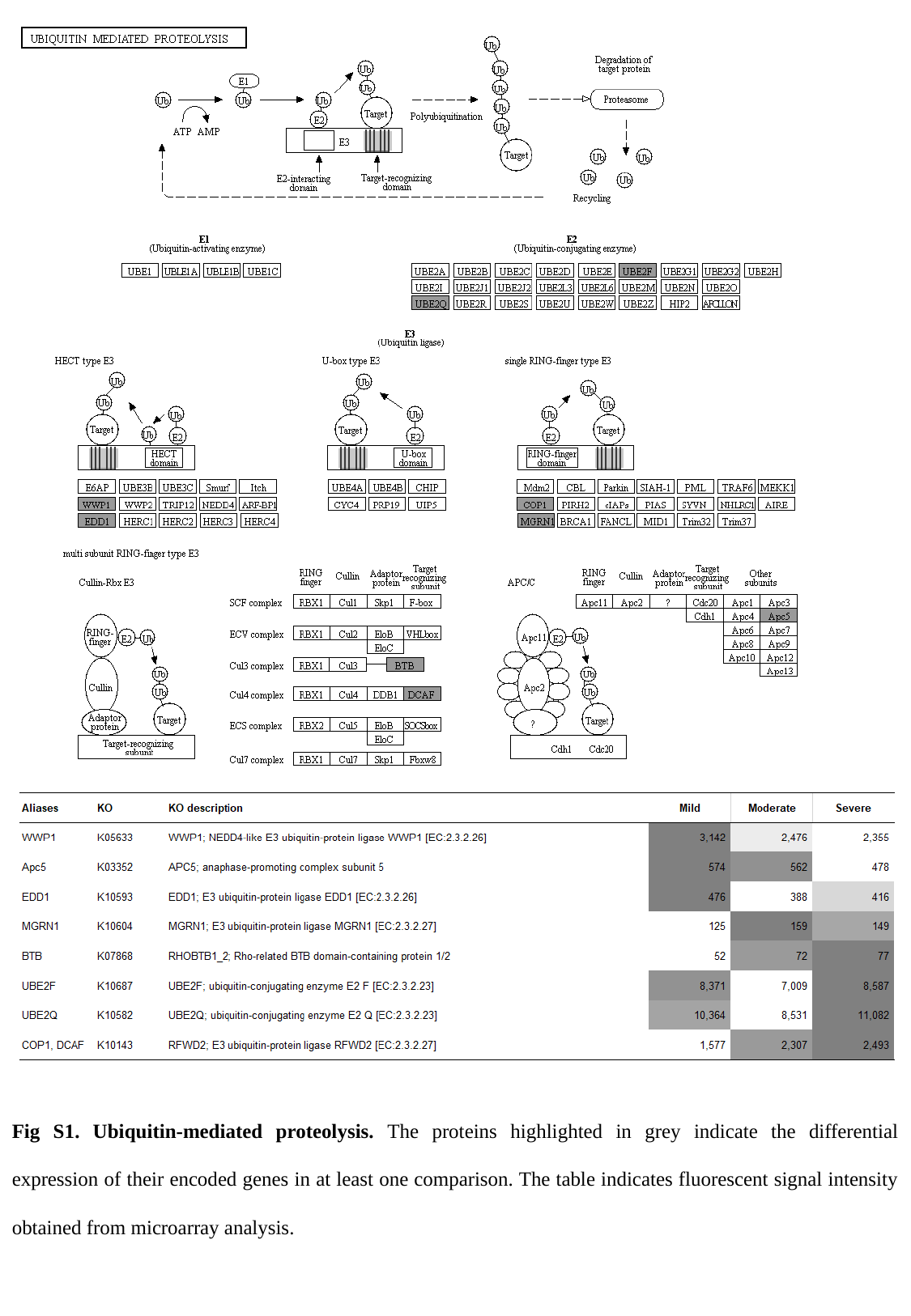

Fig S1. Ubiquitin-mediated proteolysis. The proteins highlighted in grey indicate the differential expression of their encoded genes in at least one comparison. The table indicates fluorescent signal intensity obtained from microarray analysis.

## Slide 2
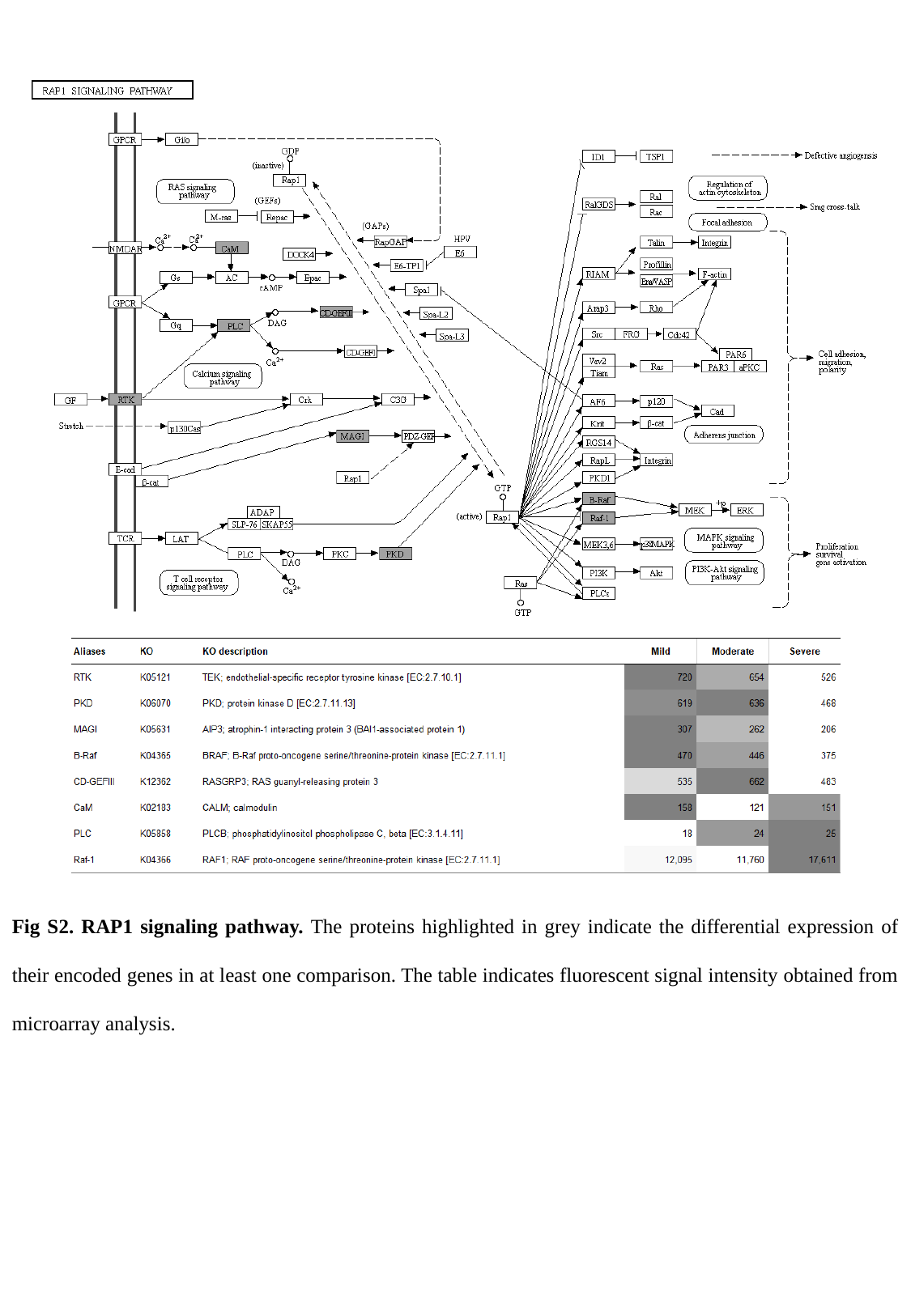

Fig S2. RAP1 signaling pathway. The proteins highlighted in grey indicate the differential expression of their encoded genes in at least one comparison. The table indicates fluorescent signal intensity obtained from microarray analysis.

## Slide 3
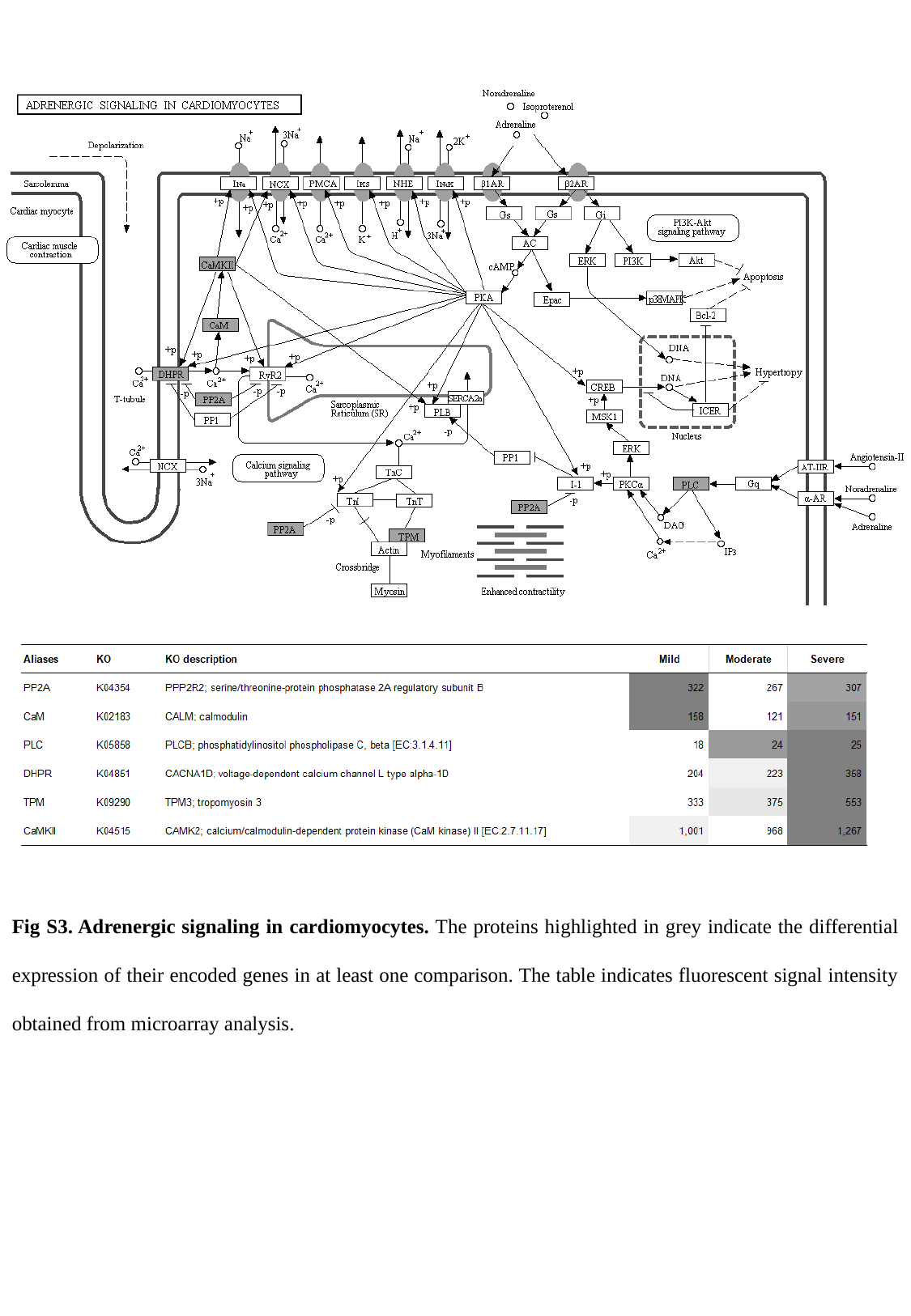

Fig S3. Adrenergic signaling in cardiomyocytes. The proteins highlighted in grey indicate the differential expression of their encoded genes in at least one comparison. The table indicates fluorescent signal intensity obtained from microarray analysis.

## Slide 4
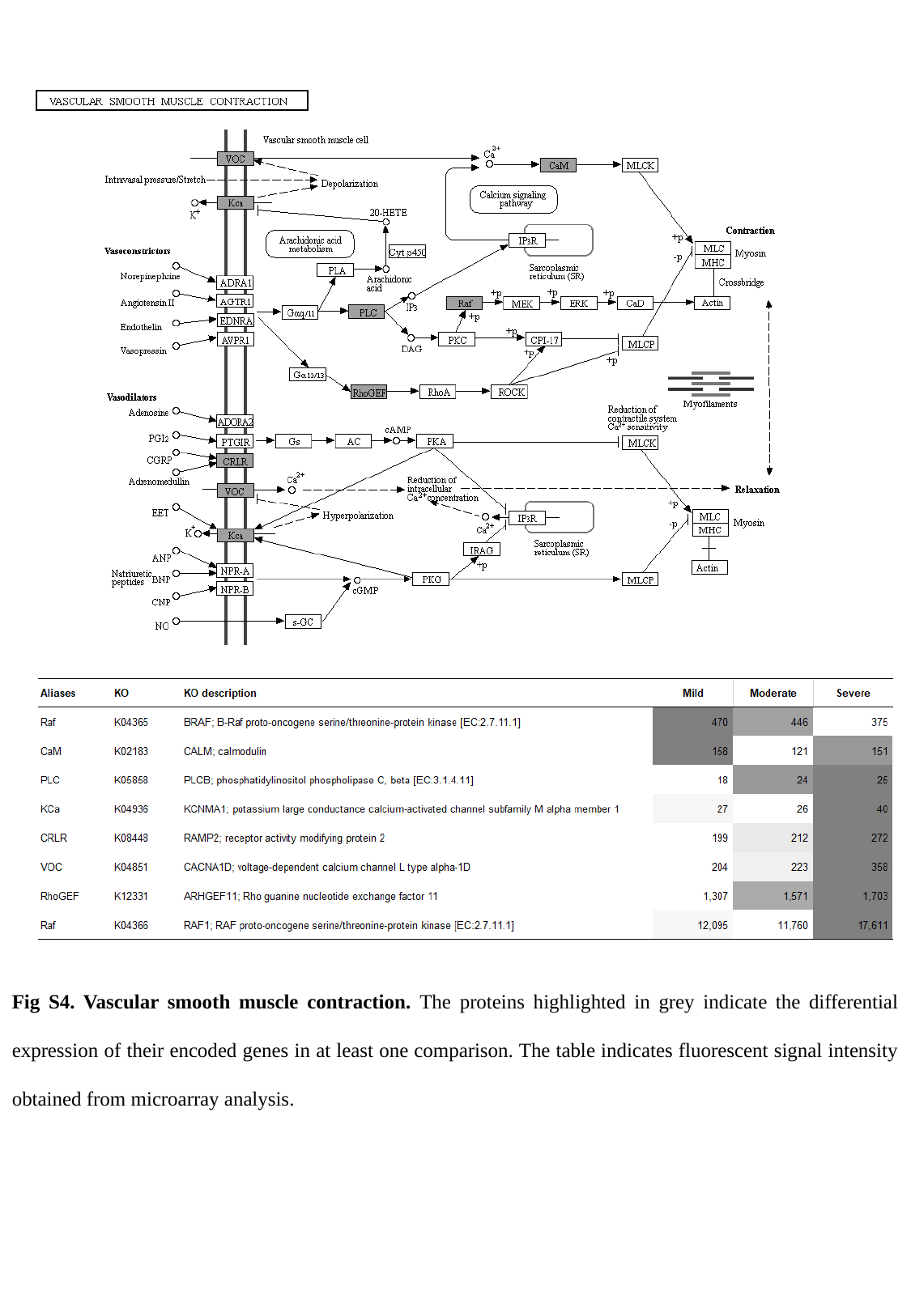

Fig S4. Vascular smooth muscle contraction. The proteins highlighted in grey indicate the differential expression of their encoded genes in at least one comparison. The table indicates fluorescent signal intensity obtained from microarray analysis.

## Slide 5
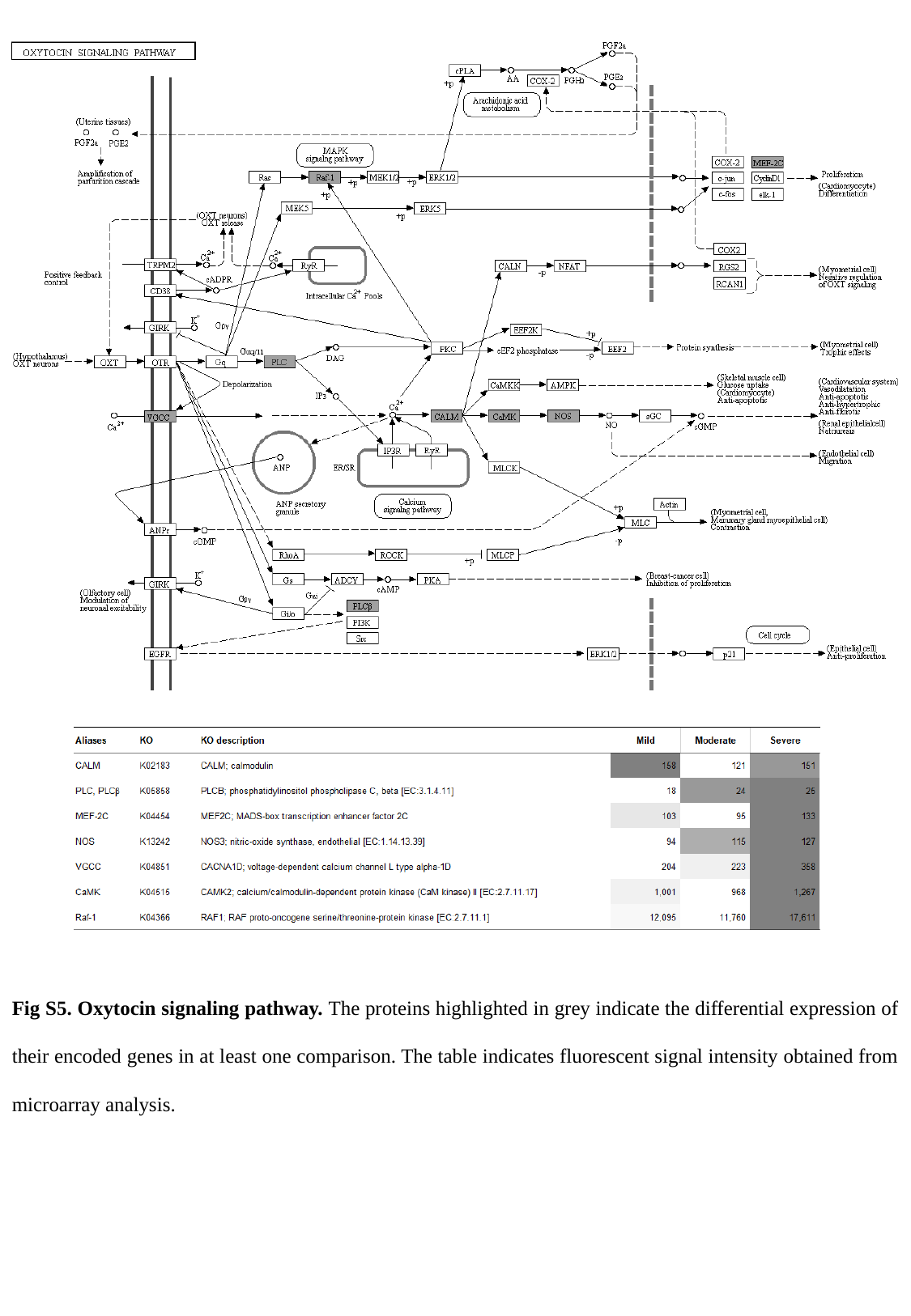

Fig S5. Oxytocin signaling pathway. The proteins highlighted in grey indicate the differential expression of their encoded genes in at least one comparison. The table indicates fluorescent signal intensity obtained from microarray analysis.
